# Immune Imprinting Across Respiratory Virus Infection, Vaccination, and Passive Immunization: Rethinking ‘Original Antigenic Sin’ in Influenza, SARS-CoV-2, and RSV

**DOI:** 10.3390/v18101155

**Published:** 2026-10-17

**Authors:** Agnieszka Szuster-Ciesielska

**Affiliations:** Department of Virology and Immunology, Institute of Biological Sciences, Maria Curie-Skłodowska University, Akademicka 19, 20-032 Lublin, Poland; agnieszka.szuster-ciesielska@umcs.pl; Tel.: +48-81-537-59-43

**Keywords:** immune imprinting, original antigenic sin, germinal center, hemagglutinin, SARS-CoV-2 spike, respiratory syncytial virus, universal influenza vaccine, back-boosting

## Abstract

Immune imprinting shapes every subsequent response to antigenically related pathogens and is now central to designing updated vaccines for influenza, SARS-CoV-2, and respiratory syncytial virus (RSV). The label “Original Antigenic Sin” has framed imprinting as a fixed liability, but recent germinal-center, B-cell repertoire, and epidemiological data show that the effect is neither uniformly harmful nor irreversible. This review distinguishes cross-reactive immune memory from immune imprinting, defined here as a preferential bias toward the first-encountered antigen. Prior exposure can provide useful cross-reactive memory, but available evidence generally does not show that first-antigen bias itself improves protection relative to an equivalent unbiased memory response. We therefore use a hypothesis-generating conceptual framework to examine how antigenic distance, conserved-epitope accessibility, and exposure interval may influence both the usefulness of recalled memory and the extent to which imprinting restricts recruitment of responses to later antigens. The framework organizes existing observations and is not intended to predict imprinting outcomes ex ante. We discuss recent quantitative models that explicitly formalize competition between memory and de novo responses as complementary, pathogen-specific approaches. Across influenza and SARS-CoV-2, early exposure can establish cross-reactive memory associated with subsequent heterologous protection, while preferential recall of the priming antigen may limit responses to novel epitopes. For RSV, the effects of maternal vaccination and passive antibodies on subsequent active immune memory remain uncertain. Immunogen engineering, schedule adjustment, adjuvant selection, and cohort-tailored strategies may broaden pre-existing memory toward conserved protective epitopes and reduce restrictive first-antigen bias.

## 1. Introduction

Respiratory virus vaccination has entered a phase in which the vaccine’s antigenic composition is updated more frequently than the vaccinee’s memory compartment can be replaced. Seasonal influenza vaccines are reformulated annually to track antigenic drift in hemagglutinin (HA) and neuraminidase (NA) [1]. Since 2022, COVID-19 vaccines have been updated roughly annually to follow the evolution of Omicron sublineages, from bivalent WT/BA.4-5 formulations to monovalent XBB.1.5-, JN.1-, and KP.2-adapted boosters [2,3]. Respiratory syncytial virus (RSV) vaccines based on the stabilized prefusion F glycoprotein were licensed for older adults and for maternal use between 2023 and 2024, creating a novel early-life passive-antibody environment whose effect on subsequent active imprinting remains unknown [4,5]. Each of these programs raises the same underlying immunological question: how does the memory established by the first vaccine or infection shape the response to every subsequent antigenically drifted immunogen?

In this review, immune imprinting refers to the preferential weighting of subsequent responses toward immune specificities established by the first antigenic exposure when the host encounters a related later antigen. This differs from pre-existing or cross-reactive immune memory, which can provide protection without a preferential bias toward the first antigen [6,7,8]. Original Antigenic Sin (OAS) denotes the narrower serological pattern first formalized by Francis in 1960: after exposure to an antigenically related but distinct influenza virus, antibody titers to the original strain remain higher than those to the later strain [9]. OAS should therefore be regarded as one potentially maladaptive manifestation of immune imprinting rather than as a synonym for the broader phenomenon [10]. Subsequent work has extended the concept of immune imprinting from serology to germinal-center dynamics, memory B-cell selection, and B-cell receptor repertoires [11,12,13]. Throughout this review, “immune imprinting” is used for the general phenomenon, whereas “OAS” is reserved for the narrower serological pattern in which responses to the first-encountered strain remain dominant after exposure to a related later strain.

Consistent with this distinction, the term Original Antigenic Sin carries a normative connotation that the broader concept of immune imprinting lacks. Cobey and Hensley reviewed evidence available through 2017 and noted that although imprinting reliably shapes the specificity and magnitude of antibody responses to drifted strains, no consistent evidence shows that individuals with pre-existing immunity are more susceptible to viral infection than immunologically naive hosts [14]. Influenza pandemic epidemiology provides strong evidence that childhood exposure history is associated with later group-matched protection. Gostic and colleagues reported reduced risk of H5N1 or H7N9 in cohorts whose inferred childhood exposure belonged to the corresponding HA group [15]. These data support durable cross-reactive immune memory, but they do not isolate an advantage of preferential first-antigen bias over an equivalent memory response without such bias. Age-shifted severity patterns during the 2009 H1N1 pandemic and the 2013 H7N9 emergence in China conform to the same mechanistic logic [16,17]. In these settings, cross-reactive memory associated with early exposure is a lifelong correlate of survival; these data do not establish a survival advantage attributable to preferential first-antigen bias itself.

The compressed evolutionary timescale of severe acute respiratory syndrome coronavirus 2 (SARS-CoV-2) has provided a real-time laboratory for this question. Individuals primed with the ancestral Wuhan-Hu-1 spike, whether by infection or by monovalent mRNA vaccination, generate cross-reactive memory B cells whose activation dominates the response to Omicron sublineages, at the expense of de novo recruitment of naive B cells to novel epitopes [11,18]. Alsoussi and colleagues used fine-needle aspiration of draining axillary lymph nodes to show that boosting with either the ancestral spike or a bivalent Beta/Delta formulation produced spike-binding germinal-center B cells that originated predominantly from pre-existing clonal lineages, whereas a monovalent BA.1-matched booster elicited rare but detectable de novo B-cell responses against novel BA.1 epitopes [12]. Yisimayi and colleagues extended this observation to repeated Omicron exposures, demonstrating that a second Omicron encounter, either through breakthrough infection or booster vaccination, expands Omicron-specific naive B cells and mitigates the imprint left by the ancestral vaccine, whereas a single Omicron exposure is insufficient [19]. Malladi and colleagues subsequently confirmed, using paired lymph-node histology and BCR sequencing, that variant boosters primarily broaden immunity by recalling memory B cells into the germinal center, where somatic hypermutation and further selection generate cross-reactive antibodies, rather than by displacing the imprinted repertoire [13]. Together, these studies show that recalled memory can mature and broaden after variant exposure, while ancestral-spike bias persists and may limit de novo recruitment to novel epitopes. This resulting breadth reflects adaptive evolution of cross-reactive memory, not, by itself, evidence that preferential ancestral bias is beneficial.

RSV occupies a distinct position in this landscape because immune imprinting is now deliberately engineered in early life. The 1966 formalin-inactivated RSV vaccine catastrophe illustrated the danger of maladaptive priming, in which antibodies against the postfusion conformation of the F glycoprotein, together with a Th2-skewed T-cell response, caused enhanced disease during subsequent natural infection [20]. Stabilizing the prefusion conformation of F resolved this problem and enabled the current generation of vaccines. Maternal RSVpreF immunization, transferred to the infant through the placenta, confers approximately 82% efficacy against medically attended severe RSV disease within 90 days of birth and 69% efficacy through 180 days, as demonstrated in the MATISSE trial and confirmed in its final analysis [4,21]. The nirsevimab monoclonal antibody, delivered at birth, provides a parallel form of passive protection without generating active immune memory, and its introduction across the 2023–2026 birth cohorts has already been associated with substantial reductions in RSV-associated hospitalization [22]. Whether the imprint established by maternal RSVpreF influences the memory repertoire of the infant’s first RSV infection is an open and actively investigated question [23].

This review examines two related but distinct consequences of immune history: the protection provided by cross-reactive memory and the preferential first-antigen bias that can shape or restrict later responses. Available studies often measure both simultaneously and therefore do not generally establish that imprinting bias itself is protective. We use the three proposed variables to organize when recalled memory is likely to remain useful and when first-antigen bias may interfere with responses to an updated immunogen.

Literature search strategy. We searched PubMed and Google Scholar from January 2020 to August 2026 using combinations of the terms ‘immune imprinting’, ‘original antigenic sin’, ‘hemagglutinin imprinting’, ‘SARS-CoV-2 immune imprinting’, and ‘RSV prefusion F’. We included studies that provided mechanistic, epidemiological, or clinical evidence directly addressing imprinting or modification. We screened the reference lists of included studies for additional sources.

## 2. Mechanistic Foundations of Immune Imprinting

**Figure 1:**
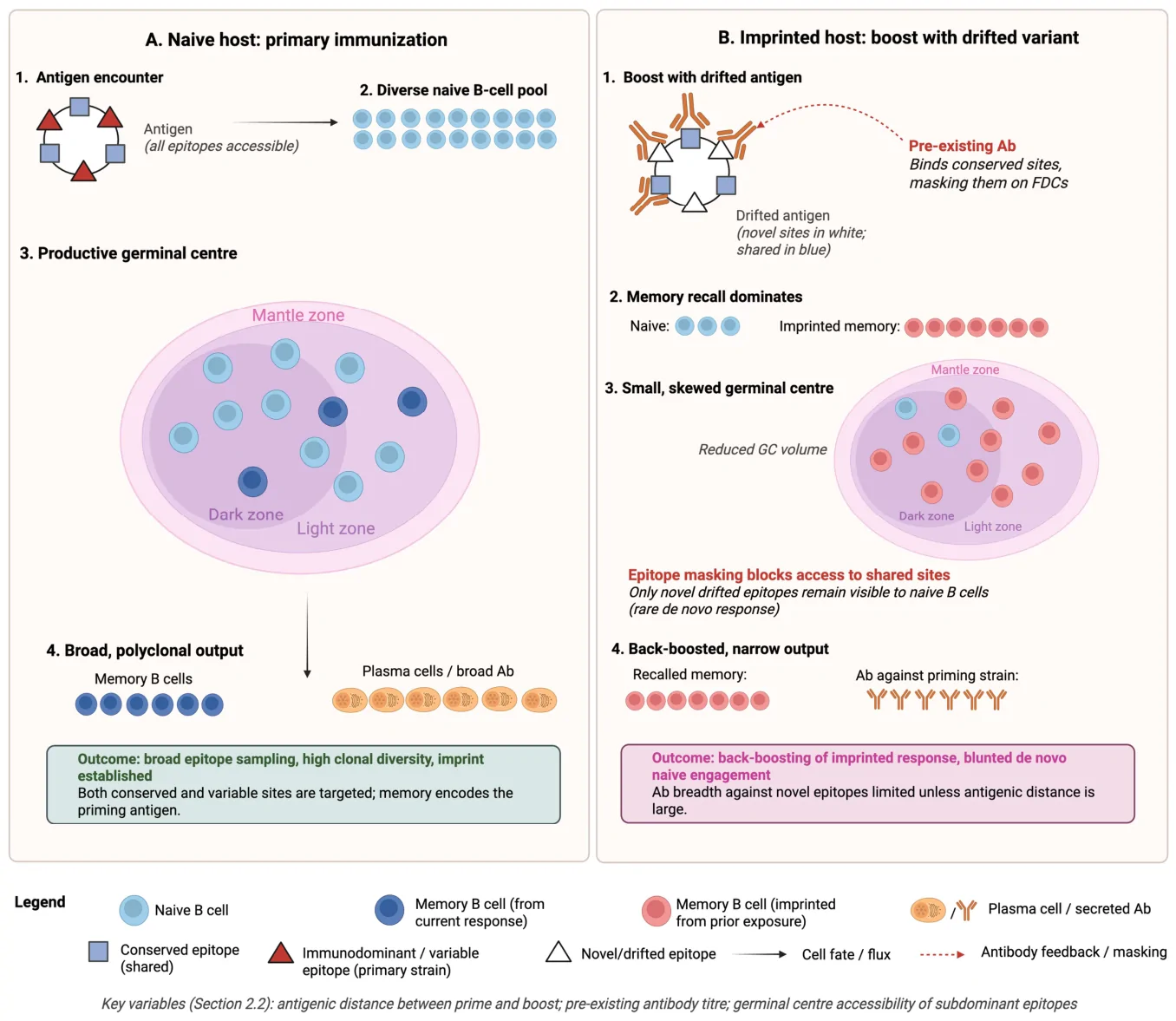
**Germinal-center outcomes in a naive versus an imprinted host.** Schematic comparison of B-cell responses after primary immunization (Panel (**A**)) and after a boost with a drifted immunogen in a previously imprinted host (Panel (**B**)). (**A**) In a naive host, all antigenic epitopes are accessible to a diverse pool of naive B cells. A productive germinal center (GC) forms with dark and light zones, supporting broad clonal expansion, somatic hypermutation, and affinity maturation. The output includes memory B cells that encode the priming antigen and plasma cells that secrete broadly reactive antibodies against both conserved and variable epitopes. (**B**) In an imprinted host, pre-existing antibodies bind conserved epitopes on the drifted antigen and mask them on follicular dendritic cells (FDCs), limiting antigen availability for naive B-cell activation. Recall of imprinted memory B cells dominates over de novo engagement of naive B cells. The resulting germinal center is smaller and skewed toward memory-derived clones. The output reflects back-boosting: reactivated memory cells and antibodies directed against the priming strain, with limited breadth against novel drifted epitopes unless the antigenic distance is large. Key variables governing the balance between recall and de novo response include antigenic distance between prime and boost, pre-existing antibody titer, and germinal-center accessibility of subdominant epitopes see Section 2.2 for mechanistic detail. Ab, antibody; FDC, follicular dendritic cell; GC, germinal center. Created in BioRender. Szuster-Ciesielska, A. https://BioRender.com/0nc134n, accessed on 18 August 2026.

Immune imprinting is a cellular and molecular phenomenon before it is an epidemiological one. Every clinical observation reviewed in the sections that follow traces back to a limited set of germinal-center and repertoire-level rules that determine whether a booster expands the pre-existing memory compartment, engages naive B cells against novel epitopes, or produces hybrid outcomes. This section assembles the mechanistic scaffold on which the virus-specific narratives depend. Four questions are addressed in turn. First, how do naive and memory B cells compete for antigen and T-cell help when a boost is administered [24,25]. Second, why is dominance of certain epitopes preserved across drifted immunogens, and what role does epitope masking by a pre-existing antibody play [7,26]. Third, what distinguishes a de novo naive response from memory recall at the level of clonal genealogy, somatic hypermutation, and antibody breadth [12,13]. Fourth, how do genetic, developmental, and structural factors of the immunogen affect the utility of recalled memory and the extent to which first-antigen bias causes interference [27,28]. Together, these mechanistic rules form a framework that we apply to influenza, SARS-CoV-2, and RSV. A schematic representation of the germinal-center dynamics discussed above is provided in Figure 1.

### 2.1. Germinal-Center Biology Relevant to Imprinting

Germinal centers (GCs) are the microanatomical sites where B-cell affinity maturation, somatic hypermutation (SHM), and class-switch recombination occur [24]. Upon first antigen exposure, activated naive B cells that receive cognate T-follicular-helper (Tfh) cell help enter the follicle and seed a GC [24,29]. Within the GC, they cycle between the dark zone, where cells divide and mutate their B-cell receptor (BCR) genes, and the light zone, where mutated BCRs compete for a limited supply of antigen displayed on follicular dendritic cells. B cells that acquire a higher-affinity BCR through SHM outcompete lower-affinity siblings for antigen capture and Tfh-cell help and are preferentially selected for further division in the dark zone. This iterative process, formalized by Victora and Nussenzweig, produces a Darwinian-like affinity-maturation trajectory that culminates in the differentiation of long-lived plasma cells and memory B cells [24,30].

The temporal choreography of these two output cell types has direct consequences for imprinting. Weisel and colleagues used a tamoxifen-inducible reporter of activation-induced cytidine deaminase expression to fate-map GC B cells in mice and demonstrated a temporal switch in GC output [31]. Early in the response, GCs preferentially generate memory B cells that carry relatively few somatic mutations and retain broad specificity. Late in the response, the same GCs shift toward producing long-lived plasma cells enriched for high-affinity, extensively mutated clones [31]. This asymmetry has two implications for imprinting. First, the memory compartment seeded by a primary immunization is populated with less-mutated clones that retain the potential to recognize drifted variants. Second, the long-lived plasma cell compartment, which sustains circulating antibody titers for years, is dominated by high-affinity clones specific for the original immunogen and can therefore mask epitopes on a subsequent drifted immunogen through pre-existing antibody feedback [31].

Hägglöf and colleagues introduced another layer of complexity, showing that ongoing GC reactions are not clonally sealed after initial seeding [25]. Using time-resolved fate-mapping in mice, they showed that up to approximately 30% of GC B cells present at late time points had entered the GC after its initial establishment, joining from the naive B-cell pool throughout the response [25]. This continuous invasion by naive B cells is functionally relevant because high-affinity pre-existing antibody produced by early plasma cells masks the immunodominant epitopes on antigen displayed by follicular dendritic cells. That masking lowers the effective affinity threshold for naive B cells with novel specificities to enter the GC and compete for T-cell help [25]. The mechanism therefore provides a cellular route by which subdominant, cross-reactive epitopes can, in principle, be engaged during a boost, provided that pre-existing antibody sufficiently occupies the dominant sites.

Whether this route is used in practice after a booster depends on the competitive dynamics between memory recall and de novo naive activation. Mesin and colleagues addressed this question by tracking the fate of memory B cells re-exposed to cognate antigen and reported that memory B cells re-entering GCs after a boost exhibit restricted clonality and limited proliferation, whereas most memory-derived response is generated by extrafollicular plasmablast differentiation rather than by re-seeding of secondary GCs [27]. Schiepers and colleagues extended this framework by using molecular fate-mapping to track serum antibody responses to repeated immunization in mice and showed that the majority of serum antibodies after a boost are derived from reactivated memory B cells that differentiate extrafollicularly into plasmablasts, with only a small fraction originating from newly formed secondary GCs [32]. In primates, however, Deimel and colleagues demonstrated that after repeated immunization, secondary GCs form more readily than in mice and that clonal expansion and diversification of GC and memory B cells continue in these secondary structures [33]. This species difference is relevant because it suggests that in humans, unlike in mice, a fraction of the response to a drifted booster can proceed through secondary GCs in which affinity maturation continues.

The relevance of these mechanistic principles to imprinting was demonstrated directly in the SARS-CoV-2 setting. Malladi and colleagues sampled draining lymph node GCs by fine-needle aspiration in adults after a bivalent ancestral-plus-Omicron BA.4-5 mRNA booster and reconstructed the GC B-cell repertoire using paired single-cell BCR sequencing [13]. Approximately 77.8% of GC B-cell clones expressed spike-binding monoclonal antibodies, of which 37.8% targeted the receptor-binding domain (RBD) [13]. One representative RBD-directed clone, designated mAb-52, neutralized all tested SARS-CoV-2 variants, including the contemporary XEC lineage, through extensive SHM accumulated during the boost-induced GC response rather than through de novo recruitment of Omicron-specific naive precursors [13]. Primary exposure can seed cross-reactive memory that serves as a substrate for continued GC-mediated maturation and cross-variant antibody generation, provided shared conserved epitopes remain structurally accessible on the updated immunogen [13]. This breadth reflects the adaptation of cross-reactive memory and does not demonstrate an independent benefit of preferential first-antigen bias. The sections on memory recall and epitope masking and on back-boosting and cross-reactive breadth examine why access to these epitopes may be limited by pre-existing antibodies and how the balance between memory recall and de novo activation of naive B cells is regulated.

### 2.2. Memory Recall and Epitope Masking

The high-affinity antibody produced during a primary immune response does not merely circulate as a passive marker of past exposure. It actively shapes the selection landscape of any subsequent germinal-center reaction against an antigenically related immunogen. When a booster is administered, the secreted antibody from long-lived plasma cells and reactivated memory B cells binds the injected antigen in the interstitium and on follicular dendritic cell surfaces, forming immune complexes and reducing the concentration of unliganded antigen available for BCR engagement in the light zone [24,30]. Because the pre-existing antibody pool is enriched for high-affinity clones directed against immunodominant epitopes on the priming immunogen, masking is not uniform across the antigen surface. Epitopes that were dominant during the primary response are most efficiently sequestered by the antibody, while subdominant and cross-reactive epitopes remain relatively more accessible for de novo BCR recognition [25].

Zhang, Meyer-Hermann, and Toellner formalized the mechanistic basis of this selective masking by combining in vivo passive-transfer experiments with an agent-based model of the germinal-center reaction to show that antibodies produced by GC-derived plasma cells re-enter GCs in an affinity-dependent equilibrium, thereby restricting antigen access on follicular dendritic cells [34]. Two effects follow. First, the masking mechanism raises the effective affinity threshold for continued selection of B cells with the same specificity as the pre-existing antibody, because these clones must now compete for a smaller pool of unbound antigen. Second, the same mechanism lowers the relative threshold for B cells with specificities against epitopes not covered by the pre-existing antibody, since these B cells face less competitive pressure at their epitopes [34]. The net consequence is that antibody feedback biases GC selection toward clones that recognize distinct or previously subdominant epitopes, providing a molecular route for diversification of the response across the antigen surface. This framework, refined by subsequent computational and experimental work, is now accepted as one of the principal cellular mechanisms by which repeated exposure to a drifting antigen can, under permissive conditions, broaden rather than narrow the antibody response [24,25].

Schiepers and colleagues provided direct experimental confirmation of this framework in the context of recall responses by tracking the composition of secondary GCs after repeated immunization with the same protein antigen in mice. They reported that antibody-mediated feedback steers recall GC B cells away from immunodominant epitopes targeted in the primary response and enables specific targeting of variant epitopes when the boosting immunogen carries such changes [35]. Tetramer-based analyses of recall GC B cells showed a marked decrease in their ability to bind antigenic sites recognized by the primary response, coupled with increased binding to alternative or variant sites. This experiment provides the closest available mechanistic parallel to the phenomenon observed after variant-adapted boosters in humans and grounds the concept of imprinting in a defined cellular process rather than in serological observation alone [35].

Direct empirical support for this framework in respiratory-virus immunity comes from the SARS-CoV-2 setting. Röltgen and colleagues compared draining axillary lymph-node biopsies from individuals vaccinated with BNT162b2 or mRNA-1273 with peribronchial lymph-node tissue from fatal COVID-19 cases [11]. Vaccination produced robust GC responses with follicular hyperplasia, abundant BCL6-positive B cells, PD-1-positive T follicular helper cells, and preserved follicular dendritic-cell networks, whereas severe primary infection was associated with disrupted follicular architecture and depleted GC compartments. Spike antigen and vaccine mRNA were detectable in GCs of vaccinated individuals for up to 60 days after the second dose, providing prolonged antigen availability for affinity maturation [11]. When these vaccinated individuals subsequently experienced breakthrough infection with the Alpha or Delta variants, their serum antibody responses retained a Wuhan-Hu-1-preferential signature rather than shifting to the variant-specific pattern observed in unvaccinated individuals with primary infection with the variant [11]. This serological imprint is consistent with the mechanistic prediction that pre-existing high-affinity antibodies mask the conserved dominant epitopes shared between Wuhan-Hu-1 and Alpha or Delta, so memory recall dominates over de novo naive activation against variant-specific epitopes. Alsoussi and colleagues later provided the cellular counterpart of this observation, showing by fine-needle aspiration that GC B cells arising after ancestral or bivalent Beta/Delta boosters derived predominantly from pre-existing clonal lineages, whereas a monovalent BA.1 booster elicited rare but detectable de novo B-cell responses against BA.1-specific epitopes [12]. Together, these data delineate the conditions under which memory recall dominates and when antibody feedback can engage novel epitopes.

### 2.3. Back-Boosting and Cross-Reactive Breadth: Benefits of Memory Rather than Imprinting Bias

Processes that accompany imprinting can, under other conditions, broaden cross-reactive memory, but such breadth is not evidence that preferential first-antigen bias is beneficial. Recall of pre-existing memory by a drifted variant not only expands the response against the newly encountered strain. It also elevates antibody titers against related strains the host has previously encountered and in some cases against strains the host has never directly encountered. This phenomenon is called back-boosting. It shows that re-exposure can amplify cross-reactive memory, but it does not, by itself, show that a preferential bias toward the first antigen is advantageous. Back-boosting and first-antigen bias may coexist and should be evaluated separately [15,17].

Fonville and colleagues established the empirical foundation for back-boosting in influenza by introducing the antibody landscape as a quantitative representation of serum reactivity across the two-dimensional antigenic map of A/H3N2 viruses spanning 43 years of evolution. They analyzed sera from 69 individuals monitored longitudinally over six years and from 225 individuals sampled before and after vaccination [36]. Two observations were central. First, after infection or vaccination, titers rose broadly and included antigenic clusters far removed from the recently encountered strain, well beyond the extent of cross-reactivity observed after primary infection in naive hosts. Second, using an antigenically advanced immunogen raised titers against both the advanced antigenic cluster and the preceding clusters that had circulated during the individual’s lifetime [36]. This second observation is the direct pharmacological expression of back-boosting and provides the rationale for using strains ahead of the currently dominant epidemic cluster in the composition of seasonal influenza vaccines. Fonville and colleagues also documented antigenic seniority, whereby titers against older strains remained systematically higher than titers against strains encountered later in life, consistent with cumulative back-boosting of memory clones directed at conserved epitopes present on multiple strains encountered throughout life [36].

The most striking epidemiological consequence of back-boosting is protection against novel hemagglutinin subtypes the host has never encountered. Gostic and colleagues examined all reported human cases of H5N1 and H7N9 avian influenza and used likelihood-based model comparison to test whether age or birth year best explained the observed distribution of severe disease. Their analysis showed that the childhood HA exposure group was the dominant explanatory factor. Individuals whose first childhood influenza A infection was with a group 1 hemagglutinin (H1 or H2) had approximately 75% lower risk of severe infection and 80% lower risk of death from H5N1, which also carries a group 1 hemagglutinin. Individuals first exposed to a group 2 hemagglutinin (H3) had comparable protection against H7N9, which also carries a group 2 hemagglutinin [15]. The protective effect was heterosubtypic and required only that the childhood exposure and the novel subtype share the hemagglutinin’s phylogenetic group. This finding supports a durable contribution of childhood exposure history and cross-reactive HA-group memory to later protection. Because the study does not compare first-biased memory with an equivalent unbiased memory response, it should not be interpreted as evidence that imprinting bias itself confers protection [15]. Follow-up work extended the same logic to seasonal influenza and showed that childhood imprinting also shapes birth-year-specific risk during seasonal H1N1 and H3N2 epidemics, with subtype-level rather than group-level cross-protection [17].

Back-boosting is not unique to influenza. In SARS-CoV-2, primary infection and mRNA vaccination both boost pre-existing antibodies against endemic human coronaviruses, particularly those targeting the conserved S2 subdomain of the OC43 spike, although these back-boosted antibodies are typically non-neutralizing [18]. With variant-adapted mRNA boosters, secondary germinal centers dominated by recalled memory B cells undergo continued affinity maturation and produce antibodies with expanded cross-neutralizing activity against multiple Omicron subvariants, so recall of pre-existing memory contributes to both breadth and specificity for the primary immunogen [13]. Taken together, these observations establish back-boosting as a general feature of recall to related respiratory-virus antigens. They show that cross-reactive memory can extend recognition across strains, but leave unresolved whether preferential first-antigen bias is beneficial, neutral, or limits responses to newly emerged epitopes. The balance between this protective effect and the epitope-masking effect described in the preceding section depends on the antigenic distance between the priming immunogen and the variant, the location of conserved versus variable epitopes on the antigen surface, and the immunodominance hierarchy of the response, all of which are examined in the pathogen-specific sections that follow.

### 2.4. Distinguishing Imprinting from Original Antigenic Sin: A Proposed Working Definition

The mechanisms described above have been discussed in the immunological literature for more than six decades, often under overlapping and inconsistently used labels. The oldest of these is original antigenic sin, coined by Thomas Francis Jr in 1960 in an address to the American Philosophical Society, in which he observed that the antibody response of an adult to a heterologous influenza infection remained dominated by antibodies specific for the strain encountered in childhood [9]. Francis framed this as an immunological “doctrine,” and the theological analogy embedded a value judgment in a purely observational finding, casting the persistence of childhood-primed antibodies as a defect. The subsequent literature amplified this framing, and for several decades, original antigenic sin was used interchangeably to refer to the serological observation, the underlying cellular mechanism, and the presumed maladaptive consequence for the host. This conflation has generated persistent confusion and, more importantly, has obscured the fact that cross-reactive memory and the first-antigen bias that may accompany it can yield different net outcomes: memory may be protective, whereas the bias may be neutral or restrictive [6,37].

In 2018, Henry, Palm, Krammer, and Wilson proposed a useful reappraisal of this literature, reviewing the accumulated evidence for and against original antigenic sin in the influenza field [37]. They concluded that the phenomenon, as originally described by Francis, refers narrowly to the serological observation that antibody titers to the first-encountered strain remain higher than titers to strains encountered later in life, even after exposure to the later strains. Yewdell and Santos extended this analysis, arguing that the term should be retained only in this restricted serological sense and that its predictive power for host outcome is context-dependent rather than uniformly maladaptive [6]. Cobey and Hensley, writing in the same period, described immune history and its consequences for influenza virus susceptibility without invoking the doctrinal language of sin, and their formulation has become the reference framework for population-level analyses of imprinting effects [14]. Arevalo and colleagues later provided direct evidence in humans that antibody responses to influenza hemagglutinin retain a stalk-directed component derived from primary infection, further supporting the interpretation that the serological pattern called original antigenic sin can coexist with broadly cross-reactive antibodies rather than only narrow strain-specific ones [28].

Building on this reappraisal, we adopt the following working definitions for the remainder of this review. Original antigenic sin is used in the narrow sense proposed by Francis and refined by Henry and Yewdell to describe a specific serological observation: after exposure to a variant of a previously encountered antigen, antibody titers to the strain that initiated the primary response remain higher than titers to the newly encountered strain [6,9,37]. This observation concerns serum reactivity, not the underlying cellular mechanism or the host’s clinical consequences. For the purposes of this review, immune imprinting refers to the preferential weighting of subsequent responses toward B- or T-cell specificities established by the first antigenic exposure. Cross-reactive immune memory refers more broadly to recallable cells or antibodies that recognize related antigens and may be protective without the dominance of the first antigen. Epitope masking and antibody feedback may generate or reinforce imprinting, whereas back-boosting can occur as a consequence of memory recall but is not, by itself, evidence of beneficial imprinting [14]. Under this definition, epitope masking and antibody feedback can generate or reinforce imprinting; back-boosting is a feature of cross-reactive memory and should be classified as imprinting only if preferential first-antigen dominance is demonstrated.

This distinction matters for three reasons. First, the language of sin implies maladaptation and is therefore not neutral. It biases interpretation toward assuming that recall is a failure mode rather than an appropriate response of a functioning immune system to a related antigen. Immune imprinting is a mechanistic descriptor of preferential first-antigen bias, whose clinical effect may be neutral or restrictive; protection should be attributed to cross-reactive memory unless an independent benefit of the bias is demonstrated. Early exposure history can be associated with broad heterosubtypic protection, whereas imprinting can also produce restricted recall that fails to engage variant-specific epitopes. Available observational studies generally cannot determine whether preferential first-antigen bias contributes independently to protection [15,17,38]. Second, the distinction clarifies the experimental target. A serological observation of a persistent titer to the priming strain does not, by itself, indicate that the recall response has failed or that vaccine composition should be changed [6,37]. The relevant question is whether the recall response contains cross-neutralizing activity against the currently circulating variant, and this question requires cellular and functional measurements rather than titer ratios alone [11,12,13]. Third, the distinction has direct implications for vaccine communication. Public and policy audiences frequently interpret original antigenic sin as evidence that repeated vaccination is harmful, particularly amid misinformation about seasonal influenza and SARS-CoV-2 boosters. George and colleagues systematically analyzed English and Italian YouTube videos and news content from 2019 to 2022 and reported that original antigenic sin was among the immunological concepts most consistently associated with anti-vaccine framing in the English-language video corpus, with the term invoked to argue that repeated boosting would dampen rather than enhance protection [39]. This observation illustrates the practical consequences of imprecise terminology and reinforces the need to clearly distinguish the neutral mechanistic descriptor from the value-laden doctrinal label. The mechanistic reality documented in this section is more nuanced. Imprinting establishes the substrate on which subsequent responses are built, and vaccine strategy should be designed to work with this substrate rather than against it. The remainder of this review examines how these principles apply to influenza, SARS-CoV-2, and respiratory syncytial virus, and it identifies the design choices that maximize protective breadth while minimizing detrimental narrowing of the response.

## 3. Influenza: The Historical Laboratory of Imprinting

Influenza is the disease in which immune imprinting was first recognized as a distinct feature of the human antibody response. It is also the pathogen system in which every core concept examined in Section 2 was initially observed, characterized, and tested against epidemiological data. Francis and colleagues formulated the doctrine of original antigenic sin from observations in Michigan cohorts vaccinated with monovalent influenza strains, and later work extended these observations across the pandemic transitions of 1918, 1957, 1968, and 2009 [6,7,9,37]. Because the influenza A virus undergoes continuous antigenic drift and periodic antigenic shift, each generation of human hosts is imprinted by a different HA-NA constellation. Serosurveys, birth cohort mortality analyses, and prospective vaccination studies have therefore accumulated over seven decades of evidence on how a first exposure shapes protection, susceptibility, and vaccine responses across the entire lifespan.

**Figure 2:**
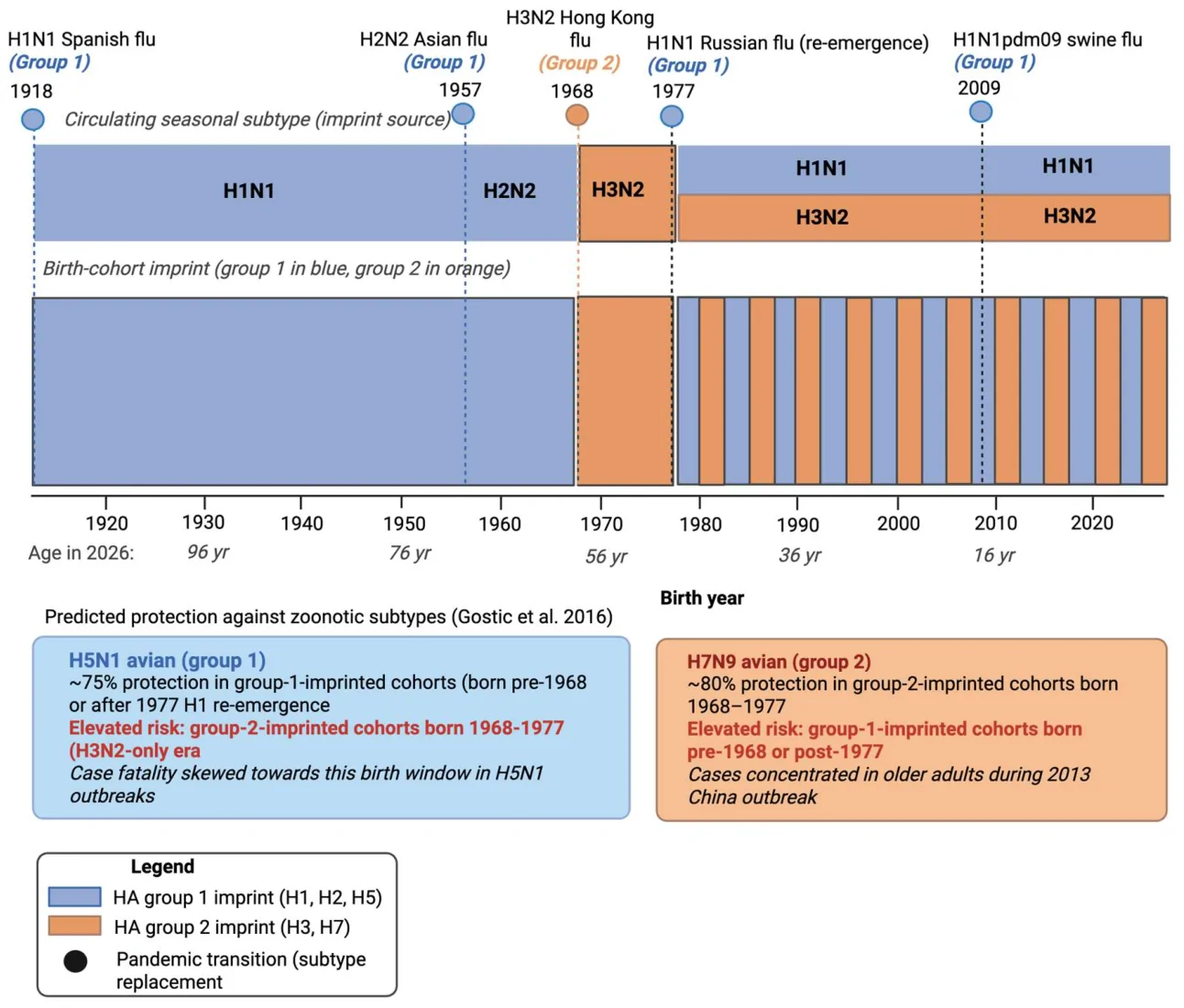
Birth-cohort imprint map for influenza HA groups and pandemic transitions, 1918–2026**.** The horizontal axis represents birth year from 1918 to 2026. Pandemic transitions (colored circles) mark the introduction of a new HA subtype into the human population: 1918 H1N1, 1957 H2N2, 1968 H3N2, 1977 H1N1 re-emergence, and 2009 H1N1pdm09. The top band shows the seasonal subtype circulating in each era, which determines the childhood imprint of contemporaneous birth cohorts. The main band shows the resulting imprint distribution across cohorts, colored by HA group (group 1 in teal, encompassing H1, H2, H5; group 2 in terra, encompassing H3, H7). During eras of subtype co-circulation (1977–2026), imprint assignment depends on which subtype caused a given child’s first exposure. The bottom panels summarize predicted protection against zoonotic H5N1 (group 1) and H7N9 (group 2), based on Gostic and colleagues [15]. Cohorts first exposed to the matching HA group show substantially reduced risk, an association consistent with protective cross-reactive memory but not proof that first-antigen bias is beneficial. The 1968–1977 birth window is uniquely vulnerable to H5N1 because it is the only era of purely group-2 (H3N2) exposure. HA, hemagglutinin. (Created in BioRender. Szuster-Ciesielska, A. https://BioRender.com/hzfles1) accessed on 18 August 2026.

The evidence on influenza is organized around four interconnected themes: historical pandemic transitions, birth-cohort and hemagglutinin-subtype effects, imprinting associated with repeated vaccination, and implications for seasonal and broadly protective vaccine design. Table 1 summarizes the principal mechanistic and epidemiological studies, while Figure 2 aligns the hemagglutinin phylogeny with the corresponding birth-cohort imprint map.

The influenza evidence is important to the review as a whole because it establishes what can be demonstrated in a well-characterized viral system. Every argument developed in later sections for SARS-CoV-2 and RSV is anchored, explicitly or implicitly, in the influenza precedent.

### 3.1. From Francis 1960 to the Modern Era

Original Antigenic Sin was defined by influenza vaccination studies showing that adults frequently developed higher antibody titers against strains encountered in childhood than against the contemporary vaccine strain. Davenport, Hennessy, and Francis demonstrated that age-specific hemagglutination-inhibition profiles reflected the influenza viruses circulating during each cohort’s childhood, and Francis subsequently formalized this observation as Original Antigenic Sin. Age-specific mortality patterns during the 1918, 1957, and 1968 pandemics linked these serological hierarchies to clinical outcomes, showing that early hemagglutinin exposure could confer either relative protection or vulnerability when a new pandemic subtype emerged [16,46,47,48].

The 2009 H1N1 pandemic provided a more directly measurable example. Using a microneutralization assay, researchers detected pre-existing cross-reactive antibody titers of 80 or higher in 39 of 115 individuals (34%) born before 1950, whereas only 4 of 107 individuals (4%) born after 1980 had titers of 40 or higher [49]. This protection was associated with structural conservation of the Sa antigenic site between the pandemic virus and early twentieth-century H1N1 strains and with lower clinical attack rates in older cohorts [50]. Conversely, some middle-aged adults generated high-titer, low-avidity antibodies associated with immune-complex formation and complement-mediated pulmonary pathology [51]. These observations established that early antigenic exposure may produce either protective or maladaptive outcomes and provided the historical basis for subsequent quantitative analyses of birth-cohort imprinting [15,17].

### 3.2. Cohort Effects, Pandemic Risk, and Imprint Hierarchy

The preceding subsection documented the birth-cohort signatures left by four pandemic transitions. This section examines the seminal study that translated these signatures into a quantitative rule for pandemic risk and discusses the rule’s consequences for pandemic preparedness planning. The organizing observation is that hemagglutinin subtypes cluster into two phylogenetic groups. Group 1 contains H1, H2, H5, and H9, and group 2 contains H3, H7, and H10. Influenza literature from 2016 to 2020 established that the inferred HA group from first childhood exposure is associated with later risk from novel avian and pandemic subtypes; this supports durable HA-group cross-reactive memory but does not establish a causal benefit of preferential first-antigen bias.

Gostic and colleagues published the definitive study on this point in Science in 2016 [15]. The authors compiled all reported human cases of avian H5N1 and H7N9 influenza and tested whether the phylogenetic group of the first seasonal HA each individual encountered, inferred from birth year and circulating strains at the time, predicted subsequent risk of severe disease. Individuals first exposed to a group-1 HA in childhood had a 75% lower risk of severe H5N1 infection and an 80% lower risk of H5N1 mortality than those imprinted with a group-2 HA, and the mirror pattern was observed for H7N9 [15]. These results support durable HA-group cross-reactive memory associated with early exposure. However, they do not establish that a preferential bias toward the first HA, rather than cross-reactive memory itself, is the causal source of protection. The childhood HA-group exposure association explained the previously puzzling age distribution of severe H5N1 cases, which concentrated in young adults born after 1968, when the circulating seasonal strain shifted from H2N2 to H3N2. Jester and colleagues reviewed the sequence of subtype replacements in detail, and their timeline confirms that H2N2 circulated as the dominant seasonal subtype from 1957 to 1968 and was displaced by H3N2 during the Hong Kong pandemic of 1968 [52]. Viboud and colleagues documented the multinational epidemiological impact of this transition and confirmed the timing of H2N2 replacement in all monitored countries [48]. The birth-year model of Gostic recovered the same pattern in both directions because H7N9 cases were concentrated among older adults who were imprinted with the group-1 H1N1 or H2N2 strains that circulated before 1968 [15]. The 2016 analysis therefore provided the first quantitative estimate of the association between childhood HA-group exposure and later group-matched protection against pandemic-scale disease and established that the HA phylogenetic group, rather than the individual strain, is the operative antigenic unit of imprinting.

The Gostic analysis is strong evidence that childhood exposure history predicts group-matched protection and can inform age-stratified pandemic risk [15]. It does not distinguish the benefit of first-antigen bias from that of cross-reactive memory; the relative contributions of imprinting bias, conserved-epitope memory, and later exposures therefore remain unresolved. A pandemic of a group-2 avian subtype, such as H7N9, would place the greatest burden on adults born before 1968, whereas a pandemic of a group-1 avian subtype, such as H5N1, would place the greatest burden on adults born after 1968 [15]. Vaccine strategies for pandemic preparedness should be evaluated against this cohort structure, because a universal H5 or H7 vaccine that succeeds in one age group may perform differently in another. Flannery and colleagues documented an operational example of this problem, reporting that VE of the 2015–2016 influenza vaccine against A(H1N1)pdm09 was only 22% among adults born during 1958–1979, compared with 61% for all other birth cohorts combined, an effect they attributed to imprinting by USSR/77-like H1N1 strains [53]. Arevalo and colleagues generalized this observation, showing that VE varies with both age and birth year in a manner consistent with early-life imprinting to the seasonal subtype [8]. The childhood HA-group association is large enough to dominate the age distribution of severe cases, but the relative contributions of cross-reactive memory, preferential first-antigen bias, and later exposures remain unresolved [15]. Gostic and colleagues extended this framework in 2019 to seasonal H1N1 and H3N2 epidemics, reporting that the same HA-group logic predicts birth-year-specific risk during seasonal transmission, with smaller effect sizes consistent with the shorter antigenic distances between successive seasonal strains within a group [17].

### 3.3. Repeat Vaccination and the Negative-Interference Literature

The preceding subsections addressed imprinting by natural infection. The influenza vaccine platform introduces a second layer of complexity because most adults have been vaccinated many times over their lifetimes, and each vaccination adds to the immunological history that shapes the response to the next season. Epidemiological studies have repeatedly detected reduced vaccine effectiveness (VE) in individuals who received the vaccine in the prior season, and the magnitude of the effect varies by subtype and by the antigenic distance between successive vaccine strains [44,54,55]. The phenomenon has been variously attributed to imprinting, to the antigenic distance hypothesis, originally formalized by Smith and colleagues in 1999 [56], and to antibody feedback mechanisms modeled by Zarnitsyna and colleagues, in which pre-existing antibody masks or clears the vaccine antigen before it can prime a broader response [57]. These explanations are not mutually exclusive.

The single most influential empirical treatment of the ADH is the analysis by Skowronski and colleagues of Canadian A(H3N2) surveillance from 2010–2011 to 2014–2015 [44]. The ADH, as formulated by Smith and colleagues, predicts that the prior-season vaccine (v1) will interfere with the current-season vaccine (v2) only when the antigenic distance between v1 and v2 is small (v1 approximately equal to v2) but large between v1 and the current epidemic strain (v1 not equal to e) [56]. Skowronski and colleagues observed exactly this pattern. In 2014–2015, when v1 and v2 were antigenically identical but the epidemic strain had drifted, VE was 65% in individuals vaccinated only in the current season and negative 33% in individuals vaccinated in both seasons, a difference of nearly 100 percentage points [44]. No interference was observed in seasons when v1 differed from v2 or when v1, v2, and e were all antigenically similar [44]. Independent replication in European I-MOVE surveillance networks has since detected concordant, ADH-consistent patterns for A(H3N2) across multiple seasons [58], and the ADH remains the most parsimonious explanation for the H3N2-specific interference signal.

McLean and colleagues had earlier documented the same phenomenon over a longer time window in the US Marshfield cohort [54]. Using eight consecutive seasons of vaccine-effectiveness data, the authors reported that current-season VE against A(H3N2) was 65% (95% CI, 36–80) among individuals with no prior vaccination in the preceding five years, but only 24% (95% CI, 3–41; *p* = 0.01) among those with a history of frequent prior vaccination [54]. The pattern was mirrored for influenza B, with 75% VE in the no-prior-vaccination group versus 48% in the repeatedly vaccinated group [54]. These results established that repeat vaccination is associated with lower current-season protection against H3N2 and B, that the effect accumulates with the number of prior seasons rather than being determined solely by the immediately preceding season, and that it is not confined to a single season or vaccine formulation.

The systematic review by Jones-Gray and colleagues consolidated the evidence base and provided the current best summary estimates [55]. The authors screened 4979 publications, included 83 in the systematic review and 41 in meta-analyses, and reported that the absolute difference in VE for individuals vaccinated in both the current and previous seasons, compared with the current season only, was negative 9% for A(H1N1)pdm09, negative 18% for A(H3N2), and negative 7% for influenza B [55]. The A(H3N2) effect was the largest and most consistent across studies [55], in line with the ADH prediction that antigenic drift is fastest for H3N2 [53]. Crucially, the same meta-analysis also reported that VE for individuals vaccinated in both seasons remained higher than for unvaccinated individuals across all subtypes and lineages [55]. The practical implication is that repeat vaccination attenuates current-season protection but does not eliminate it. The authors concluded that the evidence does not warrant a change in annual vaccination recommendations. The mechanism of the observed attenuation remains ambiguous, because ADH, imprinting, and antibody feedback all predict a similar direction of effect, and the available epidemiological designs cannot cleanly separate their contributions.

Evidence that pre-existing cross-reactive memory can be recruited to broaden vaccine responses despite persistent imprinting when the right adjuvant is used has further complicated interpretations of the negative-interference literature. In a 2026 Nature Communications study, Alcocer Bonifaz and colleagues characterized H5N1 cross-reactive antibody landscapes in individuals with defined imprinting histories and assessed the effect of AS03-adjuvanted pandemic H1N1 vaccination in a population partially naive to pH1N1 [59]. Pre-vaccination, H5N1 cross-neutralizing antibodies were detected in 91% (121/133) of participants in the 2009 pH1N1/AS03 cohort and in 100% (44/44) of participants in the 2023 cohort [59]. A single dose of the AS03-adjuvanted pH1N1 vaccine increased H5N1 cross-neutralizing antibodies by 3.7-fold and abolished pre-existing differences between birth-year cohorts, effectively overcoming the imprint that had otherwise limited the H5N1-directed response in group-2-imprinted individuals [59]. A non-adjuvanted seasonal vaccine produced only a marginal 1.3-fold increase, and recent seasonal vaccination blunted the AS03-boosted response [59]. The findings of Alcocer Bonifaz and colleagues are therefore an important counterweight to the pessimistic reading of the repeat-vaccination literature. They show that childhood imprinting is not a fixed ceiling on future breadth and that the choice of antigen and adjuvant can recruit pre-existing memory B cells toward conserved epitopes that were not targeted by the original imprinting exposure.

The combined implication of the three studies is that repeat annual vaccination has measurable but modest costs to current-season VE, driven mainly by antigenic distance effects for A(H3N2), and that the imprinting substrate on which those costs act is not immutable [44,54,55,59]. Adjuvanted platforms and antigen-choice strategies can redirect the imprinted response toward broader cross-reactivity, at least under conditions of partial pre-existing immunity and appropriate priming [59]. This dual reading of the imprinting literature will be central to the discussion of universal influenza vaccine design below, because a candidate universal vaccine must exploit durable cross-reactive memory established by prior exposure while avoiding negative interference from repeated administration of antigenically similar strains.

### 3.4. Toward a Universal Influenza Vaccine: Imprint-Aware Strategies

The preceding sections established that imprinting is durable, can bias future vaccine responses toward variable head epitopes, and that repeated annual vaccination sometimes attenuates current-season protection through a combination of antigenic distance effects, imprinting, and antibody feedback [44,54,55,56,57]. Any credible universal influenza vaccine strategy must therefore be imprint-aware, meaning it either redirects recalled memory toward conserved epitopes that pre-existing antibodies do not efficiently mask or bypasses the imprinting hierarchy with antigens that pre-existing memory has not previously encountered. The imprint-aware universal vaccine pipeline is now dominated by chimeric hemagglutinin (cHA) strategies developed largely by the Krammer and Palese groups, computationally optimized broadly reactive antigens (COBRAs) developed by the Ross group, and recombinant multivalent HA constructs derived from genetically distinct parental viruses. The current state of the field is compiled in the Universal Influenza Vaccine Technology Landscape, which is maintained by CIDRAP as part of the Influenza Vaccines R&D Roadmap, and the 2026 edition of that landscape provides the anchor reference for the technical descriptions that follow [60].

The chimeric hemagglutinin approach exploits pre-existing stalk-reactive memory while redirecting or minimizing first-antigen bias rather than fighting it. Krammer and colleagues designed HA constructs in which the immunodominant globular head is replaced by an exotic head from an avian subtype to which humans have no pre-existing memory, while the conserved stalk remains derived from a group-1 or group-2 human strain [41]. Sequential immunization with two or more chimeras carrying different exotic heads on the same stalk therefore presents naive head epitopes with each dose, while repeatedly boosting the stalk. Pre-existing head-specific antibodies do not mask the exotic head, and the memory B cells recruited with each boost recognize the conserved stalk. The 2013 preclinical work by Krammer and colleagues demonstrated that this strategy elicits broadly protective stalk-specific antibodies in mice against divergent H1, H5, and H6 challenge viruses [41]. The concept was subsequently translated into a first-in-human, observer-blind, randomized, placebo-controlled phase 1 trial (NCT 03300050). Bernstein and colleagues reported interim immunogenicity results in 2020 [42], and Nachbagauer and colleagues published the full analysis in Nature Medicine in 2021 [43]. In that trial, 66 adults aged 18–39 years received one of several regimens combining a live-attenuated cHA priming dose with an AS03-adjuvanted or non-adjuvanted inactivated cHA boost three months later. Vaccination was safe and induced broad, strong, durable, and functional antibody responses targeting the conserved, immunosubdominant stalk of the hemagglutinin [43]. The AS03-adjuvanted regimen was the most immunogenic, consistent with the Alcocer Bonifaz observation that AS03 preferentially recruits pre-existing cross-reactive memory B cells [43,59]. Follow-up mechanistic work by Aydillo and colleagues characterized the transcriptomic signature that precedes cHA-induced anti-stalk antibody induction, providing an early correlate that can be used to screen next-generation cHA constructs [61].

The Krammer strategy has since been extended to two related platforms now in the CIDRAP 2026 landscape [60]. The first is a cHA construct delivered via a live-attenuated influenza vaccine backbone with a deleted or truncated NS1 gene (cHA-ΔNS1-LAIV). NS1 is the primary influenza-encoded interferon antagonist, and its deletion abolishes viral suppression of the host type-I interferon response. Consequently, the vaccine virus becomes replication-deficient and self-adjuvanting in interferon-competent tissue [62,63]. This platform preserves the intranasal mucosal priming advantage of LAIV while enhancing innate activation of stalk-directed B cell responses. Rathnasinghe and colleagues have shown in a preclinical study that sequential intranasal immunization of mice with cH8/1-ΔNS1-LAIV followed by cH11/1-ΔNS1-LAIV induces broad serum and mucosal antibodies against the group-1 stalk, antibody-dependent cellular cytotoxicity, and protection against both seasonal H1N1 and a lethal heterologous highly pathogenic avian H5N1 challenge [62]. The second is an mRNA-encoded cHA platform, in which nucleoside-modified mRNA in lipid nanoparticles delivers the chimeric antigen without the constraints of viral rescue and enables rapid iterative design. Styles and colleagues have reported protective immunity and bone marrow plasma cell induction in rhesus macaques after mRNA-cHA immunization, and independent groups have demonstrated broad protection with combined cHA plus M2 mRNA cocktails in animal models [64,65]. Both platforms remain in preclinical or early clinical development according to the 2026 landscape [60].

Recombinant multivalent HA constructs offer an alternative imprint-aware approach that does not require sequential exotic-head immunization. The TX98-129 construct, which is described in the CIDRAP 2026 landscape, is a recombinant influenza virus expressing chimeric HA-129 derived by molecular breeding (gene shuffling) from the HAs of four genetically distinct swine influenza A(H1N1) viruses with a documented history of zoonotic transmission [60]. The rationale is that a single antigen assembled from four evolutionarily divergent parental HAs will present a broader array of conserved and semi-conserved epitopes than any single natural HA, and will therefore elicit protective immunity against genetically divergent H1 lineages regardless of the recipient’s imprinting history [60]. TX98-129 is currently at the preclinical or early clinical stage [60].

The COBRA approach employs a distinct design philosophy. Rather than physically shuffling parental HA genes, the Ross group applied a layered consensus algorithm to circulating HA sequences to compute a synthetic, ancestor-like antigen that maximizes coverage of the antigenic space occupied by past and current strains. The original COBRA H5N1 construct, published by Giles and Ross in 2011, elicited broadly reactive antibodies in mice and ferrets against diverse H5N1 clades [66]. Subsequent COBRA constructs targeting seasonal H1N1, H3N2, and influenza B lineages have been shown to elicit broadly neutralizing antibodies that recognize conserved head and receptor-binding-site epitopes distinct from those targeted by wild-type HA vaccines [67,68]. A 2023 study by Ross and colleagues extended COBRA methodology to influenza B and demonstrated that VLP-displayed COBRA IBV-HA elicited protection against a broad panel of Victoria- and Yamagata-lineage viruses in ferrets [69]. Inactivated whole-virus vaccines expressing COBRA HA have subsequently been shown to elicit long-lived plasma cell responses and durable cross-reactive antibodies in preclinical models [70]. Mechanistically, COBRAs appear to preferentially recall memory B cells with broadly neutralizing activity, suggesting that the pre-existing imprinted repertoire already contains cross-reactive clones that a wild-type HA vaccine fails to expand [68].

A common design principle underlies the platforms discussed above. Each strategy accepts the reality of imprinting and engineers the antigen to exploit it rather than to override it. In the cHA platform, recalled memory is deliberately redirected toward the conserved stalk by masking the immunodominant head with successive exotic-avian heads, so that pre-existing head-specific antibodies do not interfere with the boost [41,43]. Recombinant multivalent constructs such as TX98-129 take a different approach, in which molecular breeding of four zoonotic H1N1 parental HAs produces a mosaic head antigen whose single exposure engages a broader epitope repertoire than any natural HA [60]. The COBRA family occupies a further design space, in which a computed consensus antigen preferentially recalls memory B cells whose receptors already carry broad cross-reactivity within the imprinted repertoire [68]. None of these candidates has yet been licensed, and all remain investigational according to the 2026 CIDRAP landscape [60]. The landscape lists over 100 candidates in various stages of development. The field’s evolving consensus is that the eventual universal or broadly protective vaccine will likely combine imprint-aware antigen design with adjuvants such as AS03 that preferentially recruit cross-reactive memory, and with delivery platforms such as mRNA that permit rapid iteration against emerging antigenic variants [43,59,60,64]. These design principles are also relevant to pandemic preparedness for H5N1 and H7N9 because imprinting by group 1 or group 2 seasonal influenza viruses shapes the baseline cross-reactive repertoire in the human population.

## 4. SARS-CoV-2: Imprinting Under the Microscope of Variant Evolution

The influenza literature described above took six decades to develop from Francis’s observation in 1960 to the current mechanistic and clinical picture, because seasonal influenza generates antigenic novelty on a timescale of years and because most humans acquire only one or at most two exposures per season [9,71]. SARS-CoV-2 compressed that timescale by two orders of magnitude. Between late 2019 and the end of 2022, the global population progressed through the ancestral Wuhan-Hu-1 lineage, the antigenically distinct variants of concern Alpha, Beta, Gamma, and Delta, and the antigenic shift represented by the Omicron sublineages BA.1, BA.2, BA.4/5, BQ.1, and XBB [72,73]. Over the same interval, most of the vaccinated global population received one or more doses of a first-generation vaccine encoding the ancestral Wuhan-Hu-1 spike, and many were subsequently exposed to Omicron and its descendants either by infection or by variant-updated boosters. This convergence of a rapidly evolving antigen with a densely sampled, molecularly resolved human immune response made SARS-CoV-2 the first pathogen for which imprinting could be characterized in real time at the level of individual germinal centers, memory B cell clones, and plasma cell repertoires [11,18,74].

The core observations of this section map directly onto the mechanistic framework developed above. Ancestral-spike priming, whether by infection or by first-generation mRNA vaccination, established a memory B cell pool oriented toward Wuhan-Hu-1 spike epitopes. Subsequent exposure to Omicron variants preferentially recalled these pre-existing cross-reactive clones through the same immunodominance and epitope-masking mechanisms that shape imprinted recall in the influenza system, rather than generating de novo naive B cell responses against Omicron-specific epitopes [38,74,75,76]. This ancestral-cross-reactive recall provided a broad protective umbrella against pre-Omicron variants of concern, but it also blunted the variant-specific component of the response to Omicron itself and, in the case of BA.5 and later sublineages, focused antibody pressure onto a narrower set of epitopes that facilitated convergent viral escape [77]. The evidence on SARS-CoV-2 is considered in relation to ancestral-strain imprinting, the Omicron transition, the restrictive consequences of ancestral-spike bias, the protective potential of ancestral-spike-derived cross-reactive memory, and the implications for variant-adapted boosters in the XBB and JN.1 eras.

### 4.1. Ancestral-Strain Imprinting and the Omicron Transition

Human evidence of imprinting on the SARS-CoV-2 spike predates both the emergence of Omicron and the rollout of mRNA vaccines. Aydillo and colleagues profiled the early humoral response in hospitalized COVID-19 patients during the first pandemic wave and quantified pre-existing antibodies to the endemic seasonal coronaviruses OC43, HKU1, and 229E [18]. They observed strong back-boosting of antibodies to conserved regions of the OC43 and HKU1 betacoronavirus spike proteins during acute SARS-CoV-2 infection, and this back-boost correlated negatively with the magnitude of the de novo IgG and IgM responses against the SARS-CoV-2 spike and nucleocapsid [18]. The imprint of prior seasonal coronavirus exposure therefore biased the primary response to SARS-CoV-2 toward the conserved S2 subunit, which is antigenically shared across betacoronaviruses, and away from the more variable and neutralization-relevant receptor-binding domain (RBD) of S1 [18]. This is the earliest direct human evidence that imprinting can operate across coronavirus species and that it can be measured within the first weeks of a novel pandemic exposure.

Röltgen and colleagues extended this framework to the vaccine era by comparing antibody breadth and lymph-node germinal-center biology in convalescent and mRNA-vaccinated individuals [11]. Their central finding refined the received view of imprinting. Contrary to the naïve prediction that natural infection samples a broader spectrum of spike epitopes than a vaccine encoding a single antigen, they observed that antibody breadth against SARS-CoV-2 variants was lower after infection than after any of the vaccines evaluated and improved only over several months [11]. Two mechanisms explained this asymmetry. First, severe COVID-19 disrupted lymph-node germinal centers, in part through the loss of Bcl-6-expressing T follicular helper cells previously reported by Kaneko and colleagues. By contrast, mRNA vaccination stimulated robust, persistent germinal-center reactions that remained detectable up to eight weeks post-vaccination in some individuals and contained vaccine mRNA and spike antigen for as long as sixty days [11]. Second, viral-variant infection in previously mRNA-vaccinated individuals elicited variant-specific antibodies, but prior mRNA vaccination imprinted the serological response toward Wuhan-Hu-1 rather than the infecting variant, so hybrid exposure did not fully overwrite the vaccine-set imprint [11]. Röltgen and colleagues thus provided the first direct histological and molecular demonstration that ancestral-spike imprinting is established preferentially in the germinal-center compartment recruited by mRNA vaccination, and that this imprint constrains the variant-specific response to subsequent heterologous exposure [11].

The emergence of Omicron BA.1 in November 2021 transformed imprinting from an inference into a real-time experiment. BA.1 carried more than thirty spike substitutions relative to Wuhan-Hu-1, including a dense cluster of RBD mutations that eliminated most of the neutralizing epitopes recognized by ancestral-strain vaccine sera [75,76]. In principle, a naive immune system encountering BA.1 could either mount a de novo response to BA.1-specific epitopes or recall pre-existing memory B cells that recognized the residual conserved epitopes shared between Wuhan-Hu-1 and BA.1. Within months, multiple independent groups showed that the second pattern dominated. Kaku and colleagues profiled spike-specific B cell responses in triple mRNA-vaccinated donors experiencing BA.1 breakthrough infection [74]. They reported that the acute response was characterized by high somatic hypermutation and a strong bias toward recognition of ancestral SARS-CoV-2, with 65 to 83% of anti-RBD B cells cross-reactive with both Wuhan-Hu-1 and BA.1 and no detectable BA.1-only-specific B cells at the early time point. Park and colleagues, working with the Veesler and Corti groups, reported analogous findings in a longitudinal cohort and formalized the observation as “imprinted antibody responses against SARS-CoV-2 Omicron sublineages” [38]. They showed that Omicron breakthrough infections preferentially expanded pre-existing memory B cells against epitopes shared between Wuhan-Hu-1 and multiple Omicron variants, rather than priming naive B cells against Omicron-specific epitopes, at least up to 180 days post-breakthrough. Quandt and colleagues demonstrated that BA.1 breakthrough infection in BNT162b2 vaccinees induced neutralizing activity against BA.1 and BA.2 but not against the later BA.4 and BA.5, and confirmed that the vaccination-imprinted memory B cell pool retained plasticity sufficient to be remodeled by heterologous spike exposure while remaining anchored to conserved epitopes [75].

Reynolds and colleagues most directly demonstrated the clinical and epidemiological consequences of this imprinting pattern in a cohort of health-care workers with varying prior-exposure histories [76]. Triple-vaccinated but previously infection-naïve individuals infected during the BA.1 wave developed boosted cross-reactive antibody, T cell, and memory B cell responses against earlier variants of concern, but their response to BA.1 itself was blunted. Individuals with prior Wuhan-Hu-1 infection, followed by three vaccine doses and then a BA.1 breakthrough, showed abrogated enhancement, as their prior imprint eliminated any additional boosting of BA.1-specific immunity that the breakthrough would otherwise have provided [76]. In contrast, individuals with prior Alpha infection retained modest boosting capacity, indicating that the imprinting hierarchy depends on antigenic distance between the imprint and the current exposure rather than on exposure order alone [76]. Cao and colleagues completed the mechanistic and clinical picture, showing that once fixed, imprinted humoral immunity against the Wuhan-Hu-1 spike constrained subsequent BA.2 and especially BA.5 breakthrough responses toward non-neutralizing antibody classes [77]. This narrowing reduced the diversity of neutralizing epitope binding and focused humoral pressure onto the RBD, promoting convergent Omicron evolution at sites such as R346, K444, and F486. The imprint of ancestral-strain mRNA vaccination therefore did not merely shape individual immune responses; it acted as a population-level selective force on the virus itself.

Taken together, the Aydillo, Röltgen, Kaku, Park, Quandt, Reynolds, and Cao studies converge on a coherent picture of ancestral-spike imprinting that anchors the rest of this section. Ancestral-spike imprinting is real, measurable within weeks of first exposure, and durable for at least six months of longitudinal follow-up [11,18,38,74]. In the currently studied cohorts, ancestral-strain mRNA vaccination is the dominant imprinting event because it drives robust, persistent germinal-center reactions that natural infection often fails to sustain [11]. When previously vaccinated individuals subsequently encounter Omicron, recall of ancestral-cross-reactive memory dominates over de novo naive activation, so the initial serological and B cell response to Omicron largely boosts Wuhan-Hu-1-directed clones rather than eliciting a de novo BA.1-specific response [38,74,75]. This response combines useful cross-reactive memory with restrictive ancestral bias: the former provides cross-variant neutralization against conserved epitopes, whereas the latter blunts variant-specific responses and concentrates selective pressure. On the maladaptive side, it blunts variant-specific responses and exerts a population-level selection pressure that has demonstrably shaped subsequent Omicron evolution at the RBD [76,77]. This biological background provides the basis for examining the restrictive consequences of ancestral-spike bias and the protective potential of ancestral-spike-derived cross-reactive memory.

### 4.2. Restrictive Consequences of Ancestral-Spike Imprinting

Whether cross-reactive memory remains useful and whether first-antigen bias becomes restrictive depend on the antigenic distance between the imprinting strain and the circulating variant. When cross-reactive epitopes are shared, recall of memory B cells accelerates and broadens neutralization. When the circulating variant has diverged sufficiently to acquire new protective epitopes, recall of the imprinted repertoire can actively suppress the emergence of variant-specific antibodies that block those epitopes. The SARS-CoV-2 pandemic provided an unusually well-resolved dataset on this cost of imprinting because deep mutational scanning, structural biology, and single-cell repertoire analysis converged on the same cellular decision point: the germinal center after antigen re-encounter.

The most direct evidence that ancestral imprinting suppresses de novo responses came from monoclonal antibody repertoire analyses of Omicron breakthrough cases. Park and colleagues reported that more than 80% of Wuhan-Hu-1 RBD-directed IgG antibodies secreted by memory B cells and plasma cells from breakthrough cases cross-reacted with BA.1, BA.2, and BA.4/5 RBDs [38]. In contrast, memory B cell antibodies isolated from Omicron primary infections up to six to seven months after infection were largely restricted to BA.1 or BA.2 RBDs and did not cross-react with BA.4/5. This asymmetry indicated that prior ancestral exposure preferentially expanded existing cross-reactive B cell pools rather than priming new B cell responses against Omicron-specific epitopes [38]. Cao and colleagues extended this observation, showing that BA.5 breakthrough infection in ancestral-primed individuals reduced the diversity of neutralizing antibody binding sites and increased the proportion of non-neutralizing antibody clones relative to individuals without prior imprinting [77]. Deep mutational scanning of monoclonal antibodies elicited under these conditions showed concentrated immune pressure on only two RBD hotspots (R346T/S and K444E/Q/N/T/M), whereas BA.2-elicited antibodies from imprinting-free individuals distributed pressure across multiple hotspots [77]. The narrowed epitope diversity of the imprinted response therefore both reduced neutralization breadth and channeled viral evolution toward the same escape residues, driving convergent RBD evolution across independent Omicron lineages [19,77].

The cellular mechanism underlying this bias was clarified by studies of antibody feedback during germinal center selection. Schaefer-Babajew and colleagues studied individuals who had received two high-affinity anti-SARS-CoV-2 monoclonal antibodies before mRNA vaccination, providing a controlled model of pre-existing antibody dominance [78]. The resulting memory B cells predominantly expressed low-affinity IgM antibodies with few somatic mutations and exhibited altered RBD epitope specificity. Only 1 of 77 anti-RBD memory antibodies tested retained neutralizing activity [79]. Mouse experiments showed that germinal centers formed in the presence of these antibodies were dominated by low-affinity B cell clones, indicating two mechanisms: pre-existing antibodies lowered the activation threshold for germinal center entry, allowing abundant low-affinity clones to participate, and they directly masked their cognate epitopes, redirecting the response to subdominant sites [78]. Inoue and colleagues showed the same feedback operating in the physiological setting of a third mRNA vaccine dose, where the frequency of Omicron-reactive memory B cells increased approximately nine months after the second dose and displayed an altered epitope distribution consistent with antibody-driven redirection [79]. In mouse models, adding or depleting anti-RBD serum antibodies produced concordant increases or decreases in the frequency of Omicron-reactive germinal center and memory B cells, confirming that circulating antibody titers directly modulate the epitope focus of the recall response [79]. Wang and colleagues extended this framework to bivalent boosting and reported that even the Wuhan-Hu-1/BA.5 bivalent formulation elicited memory B cells whose serological output remained dominated by ancestral-cross-reactive antibodies, an effect they termed deep imprinting attributable to the vaccine’s ancestral component [80].

Cryo-electron microscopy and deep mutational scanning together mapped the structural consequences of this epitope-masking process. Structural analysis of the Omicron BA.1 spike bound to cross-reactive Fab fragments derived from ancestral-primed memory showed conservation of contacts at antigenic site II and the class 1/4 RBD epitopes, but not at the class 1 and class 2 sites, which are heavily mutated in Omicron sublineages [38]. Starr and colleagues generated genome-wide deep mutational scanning maps of the Wuhan-Hu-1, BA.1 and BA.2 receptor-binding domains and showed that escape from the class 3 monoclonal antibody LY-CoV1404 (bebtelovimab) was driven principally by substitutions at K444, V445 and P499, with additional escape at site 446 in the Omicron backgrounds [81]. Escape from polyclonal human sera was subsequently mapped at the level of the complete spike by Dadonaite and colleagues, whose XBB.1.5 and BA.2 libraries covered nearly all spike mutations present in XBB and BA.2.86-descended clades and localised the dominant serum-escape sites to the receptor-binding domain [82]. Malladi and colleagues subsequently showed at the tissue level that when germinal centers are re-entered after variant boosting, recalled memory B cells that acquire additional somatic hypermutation on class 1/2 RBD epitopes can generate cross-variant broadly neutralizing antibodies, exemplified by the IGHV3-66 clonotype mAb-52, which neutralized all tested SARS-CoV-2 strains, including XEC [13]. Broadening therefore remains possible, but it requires sustained germinal center activity long enough for somatic hypermutation to rescue affinity against divergent epitopes, a condition frequently not met after a single variant exposure.

Taken together, these mechanistic data reframe the imprinting cost as a competition problem within the germinal center. Ancestral-primed, high-affinity memory B cells outcompete naive precursors for antigen and T follicular helper support. The antibodies they secrete rapidly mask the dominant class 1 and class 2 epitopes on the incoming variant spike, further suppressing recruitment of new naive B cells targeting those epitopes [78,79]. The result is a narrower repertoire that is less mutated at variant-specific sites and biased toward conserved but non-neutralizing epitopes, precisely the features that both reduce individual protection and increase population-level selective pressure for convergent immune escape [19,77]. This mechanistic synthesis explains why simply removing the ancestral component from the vaccine formulation, as attempted with the monovalent XBB.1.5 booster, did not fully abrogate back-boosting, and it defines the biological targets that next-generation immunogens must displace. These restrictive effects reflect first-antigen bias; by contrast, recall directed against conserved epitopes can confer protective breadth through cross-reactive memory, without establishing that the bias is beneficial.

### 4.3. Protective Cross-Reactive Memory Despite Ancestral-Spike Imprinting

Ancestral-spike priming can generate durable cross-reactive memory against conserved spike epitopes while biasing subsequent responses toward the ancestral antigen and limiting de novo recognition of novel epitopes. The protective effects reviewed here, including back-boosting, broadly neutralizing antibodies, and hybrid immunity, demonstrate the benefits of immune memory but do not establish that preferential ancestral bias itself is advantageous [74,83].

Back-boosting to conserved epitopes exemplifies protective cross-reactive memory. The S2 subunit contains a stem-helical motif spanning residues 1146 to 1159 that is highly conserved across sarbecoviruses, merbecoviruses, and embecoviruses. Pinto and colleagues [84] isolated the monoclonal antibody S2P6 from COVID-19 convalescent donors and showed that it recognizes this stem helix and neutralizes pseudotyped viruses from three betacoronavirus subgenera by inhibiting membrane fusion. Structural analysis of the S2P6 complex demonstrated that a single antibody clone can disrupt the trimeric stem-helical bundle required for fusogenic rearrangement. Passive transfer reduced viral burden in hamsters through both neutralization and Fc-mediated effector functions [84]. Additional stem-helical bnAbs, including CC40.8 and CV3-25, share this footprint and neutralize clade 1a viruses (SARS-CoV-1, WIV1, SHC014) and clade 1b viruses (SARS-CoV-2, Pang17), with activity extending in some cases to MERS-CoV [84,85]. In parallel, the class 4 receptor-binding site harbors a cryptic conserved epitope recognized by antibodies such as S2X259, which broadly neutralizes representatives of every sarbecovirus clade tested and exhibits an escape landscape restricted to a single-residue substitution [86]. When priming with the ancestral spike elicits or expands B-cell lineages that seed such conserved epitopes, subsequent exposures to Omicron or to zoonotic sarbecoviruses back-boost cross-reactive memory rather than antigenically distinct clones. These observations support the value of conserved-epitope memory; without an unbiased-memory comparator, they do not demonstrate that preferential ancestral bias improves the response.

Hybrid immunity, defined as vaccination combined with infection in either order, produces a serological profile that is both quantitatively and qualitatively superior to either exposure alone. Bobrovitz and colleagues [87] conducted a WHO-commissioned systematic review and meta-regression of 26 studies. Hybrid immunity conferred by primary-series vaccination plus infection provided 97.4% protection against severe COVID-19 at 12 months (95% CI 91.4 to 99.2), compared with 74.6% for infection alone and lower estimates for vaccination alone in the same window [87]. Hybrid immunity demonstrates the benefit of multiple antigenically and anatomically distinct exposures. Continued affinity maturation and repertoire broadening may persist despite ancestral-spike bias and may partially overcome its restriction on de novo responses; these benefits should not be attributed to the bias itself. The advantage was not limited to severe disease. Protection against reinfection at 12 months reached 41.8% for hybrid immunity versus 24.7% for infection alone [87]. Mechanistically, hybrid immunity broadens the antibody repertoire, deepens somatic hypermutation in memory B cells, and elicits mucosal responses that vaccination alone rarely achieves. Reynolds and colleagues [76] documented that ancestral-strain imprinting can dampen recall with repeated exposures, yet in the hybrid-immunity setting, the diverse antigenic input appears sufficient to overcome this dampening and drive continued affinity maturation. The functional consequence is a serum profile with higher neutralizing titers across Omicron sublineages, a longer half-life, and enrichment for antibodies targeting conserved regions of the receptor-binding domain and the S2 subunit [87].

The implications for pandemic preparedness follow directly. If ancestral imprinting targets conserved epitopes, a population primed with the ancestral spike harbors a latent reservoir of cross-reactive memory that can be mobilized against future sarbecoviruses. Rappazzo and colleagues [88] demonstrated this principle using the engineered monoclonal ADG-2, derived from a SARS-CoV-1 survivor and affinity-matured against SARS-CoV-2. ADG-2 recognizes a conserved receptor-binding epitope that overlaps the ACE2 footprint. It neutralizes SARS-CoV-1, SARS-CoV-2, and pre-emergent bat sarbecoviruses WIV-1 and SHC014, with 50% inhibitory concentrations of 1 to 30 ng/mL, and protected mice against both SARS and COVID-19 in prophylactic and therapeutic settings [88]. The optimized derivative ADG20 (adintrevimab) retained activity against successive VOCs, including Omicron, and was authorized in 2024 as pemivibart for pre-exposure prophylaxis in immunocompromised patients. Recent cross-neutralization studies of convalescent plasma extend the principle to natural immunity. In a 2024 report, 61.65% (45 of 73) of plasma samples from COVID-19 convalescent donors cross-neutralized seven or more of eight tested sarbecoviruses, compared with only 6.74% (6 of 89) of samples from vaccinated donors. Six individual monoclonal antibodies isolated from these donors, including SCM12-61 and VSM9-12, neutralized every sarbecovirus tested [89]. Cross-reactive memory seeded by ancestral-spike priming can support universal sarbecovirus vaccine strategies, although preferential ancestral bias may still constrain responses to novel epitopes.

Two design principles emerge from this beneficial aspect of cross-reactive memory. The first is antigenic conservation: vaccines that focus the immune response on conserved regions (the S2 stem helix, the class 4 RBD-core epitope, the fusion peptide) rather than on the antigenically labile RBM will broaden useful cross-reactive memory while reducing the restrictive effects of first-antigen bias. Nanoparticle displays of stabilized S2 stem-helix immunogens and glycan-shielded RBD constructs are advancing toward this goal [84,86]. The second is exposure diversification: hybrid-immunity mechanisms indicate that combining vaccination with heterologous exposure (whether through infection or variant-matched boosting spaced beyond the antibody-feedback window) can mitigate this competitive suppression and promote continued diversification of the B-cell repertoire [76,87]. Together, these principles frame ancestral imprinting not as a fixed adversary but as a design constraint that can be redirected to broaden immunity against currently unforeseen coronaviruses.

### 4.4. Implications for Variant-Adapted Boosters in the XBB and JN.1 Eras

The rollout of variant-adapted boosters from late 2022 to 2024 turned the imprinting hypothesis into an operational vaccine policy question. Two successive design choices, first a bivalent Wuhan-Hu-1/BA.4-5 formulation and then a monovalent XBB.1.5 formulation, provided a natural experiment to assess how much of the recall response could be redirected toward contemporary antigens. Head-to-head comparisons published in 2023 and 2024 showed that the bivalent WT/BA.4-5 booster did not significantly increase Omicron-neutralizing titers compared with ancestral-only boosting, an outcome attributed to preferential recall of Wuhan-Hu-1 spike memory B cells and limited priming of Omicron-specific naive precursors [90]. The removal of the ancestral component in the XBB.1.5 formulation was therefore intended to reduce back-boosting and broaden the response toward circulating XBB and JN.1 sublineages [90]. Whether this design change would blunt imprinting became the central empirical question of the 2023 to 2024 booster campaigns.

Wrynla and colleagues addressed this question in a prospective clinical cohort and reported that immune imprinting and dosing interval jointly determined antibody responses to the monovalent XBB.1.5 vaccine [91]. Participants who had previously received three to four doses of the Wuhan-Hu-1 vaccine but declined the bivalent booster (non-recipients) showed markedly greater fold-boosting of neutralizing antibodies against XBB.1.5 (18.4-fold vs. 6.2-fold), EG.5.1 (30.9-fold vs. 7.0-fold), and JN.1 (9.2-fold vs. 3.7-fold) than bivalent recipients [91]. Non-recipients also had a longer mean interval since prior vaccination (707 vs. 361 days). Because this interval was tightly collinear with bivalent status, multivariable models could not fully separate the two contributions [91]. Critically, however, ancestral spike titers were back-boosted in both groups, providing interval-independent evidence that imprinting persisted after removal of the WT component from the formulation [91]. The data therefore show that prior antigen history and temporal spacing are jointly associated with the response to variant-adapted boosters, but this cohort cannot determine their independent contributions.

Tortorici and colleagues extended this observation by mechanistically dissecting the memory B cell compartment after XBB.1.5 vaccination [92]. They showed that the XBB.1.5 mRNA booster elicited neutralizing antibodies against contemporary variants, with responses dominated by recall of pre-existing memory B cells originally induced by the Wuhan-Hu-1 spike rather than by priming XBB-specific naive precursors [92]. Immune imprinting therefore persisted after multiple exposures to Omicron spike antigens through vaccination and infection, including after administration of the updated XBB.1.5 booster [92]. This finding paralleled independent work by Johnston, Painter, Li, and colleagues, who reported that primary and secondary XBB exposures elicited mostly cross-reactive antibodies to the ancestral spike, with XBB-specific responses arising only in individuals with the lowest pre-existing humoral immunity [93]. Together, these datasets indicated that a single exposure to a divergent variant, whether through infection or updated boosting, is generally insufficient to overwrite an ancestral-imprinted repertoire.

Complementary evidence from Yisimayi and colleagues clarified the conditions under which ancestral imprinting can be substantially mitigated. In this review, we use the term “override” operationally to denote partial mitigation of ancestral-response dominance, evidenced by a reduced ancestral-cross-reactive fraction and increased recruitment and maturation of Omicron-specific B cells, rather than the complete elimination of ancestral-derived clones or antibodies. Using both mouse models and human cohorts with defined exposure histories, Yisimayi and colleagues showed that repeated Omicron exposures, operationally defined as at least two Omicron infections or one infection plus one Omicron-based booster, reduced but did not erase ancestral imprinting and generated broad neutralization in plasma and nasal mucosa [19]. The proportion of antibodies targeting conserved but weakly neutralizing epitopes fell from more than 50% after a single BA.5/BF.7 breakthrough to approximately 20% after a second Omicron exposure, indicating a substantive epitope shift rather than a purely quantitative boost [19]. The authors interpreted these findings as evidence that Omicron-specific naive B cells recruited during the first Omicron exposure can be expanded and matured by the second, providing the cellular substrate for broadened neutralization.

Malladi and colleagues provided direct histological evidence for the underlying germinal-center dynamics by examining draining axillary lymph nodes via fine-needle aspiration after a bivalent SARS-CoV-2 mRNA booster vaccination [13]. They reported that recalled memory B cells were predominantly recruited into germinal centers, where they underwent further selection and affinity maturation. They also found that 77.8% of clones expressed spike-binding monoclonal antibodies, of which 37.8% targeted the RBD [13]. One RBD-targeting monoclonal antibody, derived from a persistent germinal-center clone using the IGHV3-66 public clonotype, neutralized all tested variants, including the recent XEC lineage, and its breadth was driven by extensive somatic hypermutation acquired within the germinal center [13]. This work established at the tissue level that variant boosters can, in favorable settings, recruit pre-existing memory B cells into productive germinal-center reactions capable of broadening neutralization, even when the initial recall response is imprinted.

Taken together, the bivalent-to-monovalent booster experience reframed the imprinting problem in operational terms. Removing the ancestral component alone did not eliminate back-boosting of Wuhan-Hu-1-targeted antibodies, and single exposures to variant antigens, whether via XBB.1.5 boosting or a first Omicron infection, generally recalled the imprinted memory compartment rather than priming a variant-specific naive response [91,92,93]. Substantial mitigation of imprinting was associated with temporally spaced, repeated exposure to the divergent antigen, conditions under which germinal-center reactions could recruit variant-specific naive B cells and mature them alongside recalled memory clones [13,19]. These findings informed the interpretation of JN.1-era immunogenicity data and set the design priorities for the next generation of variant-adapted formulations. Table 2 summarizes key mechanistic studies of SARS-CoV-2 imprinting that support the harm, override, and help framework developed in this section.

## 5. RSV: The Emerging Test Case for Imprint-Aware Pediatric and Maternal Immunization

Respiratory syncytial virus (RSV) holds a unique place in the imprinting literature. Every person encounters RSV in early childhood, and reinfection persists throughout the lifespan. The virus circulates as two antigenic subtypes, A and B, which co-circulate in most seasons and drift independently. Disease severity is age-dependent, with the highest risk of severe lower respiratory tract illness concentrated in the first six months of life [95,96]. The years 2023 to 2026 brought a coordinated intervention landscape unlike that for any previous respiratory pathogen. Two prefusion F protein vaccines were licensed for older adults, a bivalent prefusion F vaccine was authorized for maternal immunization, and a long-acting monoclonal antibody, nirsevimab, entered universal infant use in several countries [97]. These interventions create distinct antigenic and passive-antibody environments in early life. Immunobridging and serological studies indicate that active responses to subsequent RSV exposure remain detectable after passive prophylaxis, but whether maternal vaccination or nirsevimab alters the infant memory B-cell repertoire or long-term imprinting trajectory remains unknown. Their implementation therefore offers an opportunity for prospective longitudinal testing rather than evidence that imprint modification has already occurred.

RSV provides a test case for imprint-aware pediatric and maternal immunization, including prefusion F vaccination, passive protection with nirsevimab, subtype-specific cross-protection, and the mechanistic safeguards that distinguish current vaccine designs from those that failed with the formalin-inactivated RSV vaccine. Throughout, the argument is that RSV illustrates a design principle applicable to every respiratory pathogen: the conformation and site of first exposure shape the trajectory of lifelong immunity, and interventions in the first months of life must be evaluated not only for immediate protection but also for the memory landscape they leave behind [96,98].

### 5.1. Prefusion F Vaccines and Their Potential Imprinting Effects

The RSV fusion (F) glycoprotein exists in two structurally distinct conformations. The metastable prefusion state (pre-F) presents antigenic sites Ø and V at the trimer apex, which are absent from the alternative postfusion (post-F) conformation. The most potent RSV-neutralizing antibodies in convalescent and vaccinee serum target sites Ø and V, and cryo-EM and biochemical mapping in the McLellan et al. structural series established their functional dominance [99,100]. Postfusion F retains shared antigenic sites, including sites II and IV, but lacks the prefusion-specific apical sites Ø and V. Consequently, post-F-based immunogens generally elicit neutralizing responses of lower potency than those elicited by prefusion-stabilized antigens. Formalin-inactivated RSV can also induce abundant non-neutralizing, low-avidity antibodies associated with immune-complex formation [101]. When vaccines present a prefusion-stabilized antigen, such as DS-Cav1 or its clinical derivatives, the repertoire is enriched for site Ø and site V lineages with 10- to 100-fold higher neutralizing potency [99].

The catastrophic 1966 trial of a formalin-inactivated whole-virus RSV vaccine (FI-RSV) provides the paradigmatic case of maladaptive priming and establishes the biological rationale for prefusion antigen design. Formalin treatment denatured the F protein, shifting its conformation toward post-F and exposing non-neutralizing epitopes. Kim, Kapikian, Fulginiti, and colleagues [20,102,103] documented that infants immunized with FI-RSV and subsequently exposed to natural RSV developed enhanced respiratory disease, with hospitalization rates several-fold higher than in unvaccinated controls. Two deaths occurred in the trial that enrolled the youngest recipients [20]. Later mechanistic studies attributed enhanced disease to two convergent defects: a Th2-skewed cellular response driven by carbonyl-modified epitopes on the denatured antigen, and low-avidity non-neutralizing antibodies that formed immune complexes and drove complement activation in the small airways [101]. The prefusion-stabilized F platform was designed to correct both defects by preserving the native conformation of the neutralizing apex and focusing the response on high-affinity, site Ø-directed lineages [99,100]. Modern prefusion F vaccine design therefore corrects the known structural and immunological defects of FI-RSV; whether it establishes a measurably different long-term B-cell memory imprint remains to be determined.

The MATISSE phase 3 trial ([4]) is the clinical translation of this design principle. The bivalent RSVpreF vaccine, containing prefusion-stabilized F glycoproteins from RSV subtypes A and B, was administered to 7392 pregnant participants at 24 to 36 weeks of gestation. In the primary analysis, vaccine efficacy against severe medically attended RSV-associated lower respiratory tract illness in infants was 81.8% (99.5% CI 40.6 to 96.3) within 90 days of birth and 69.4% (97.58% CI 44.3 to 84.1) within 180 days of birth. Efficacy against any medically attended RSV-associated lower respiratory tract illness was 57.1% (99.5% CI 14.7 to 79.8) within 90 days and 51.3% (97.58% CI 29.4 to 66.8) within 180 days [4]. The final MATISSE analysis, published in Obstetrics and Gynecology in 2025, confirmed and refined these estimates: 82.4% (95% CI 57.5 to 93.9) at 90 days and 70.0% (95% CI 50.6 to 82.5) at 180 days against severe disease, with 55.3% (95% CI 23.8 to 74.6) efficacy against RSV-associated hospitalization at 180 days [21]. Subtype-specific efficacy estimates differed numerically: efficacy against severe RSV-B disease was 75.5% (95% CI 53.3 to 88.1), whereas efficacy against severe RSV-A disease was 52.9% (95% CI −15.1 to 82.4). However, the RSV-A estimate was based on only 17 cases in the placebo group and was statistically imprecise, with the confidence interval crossing zero. This difference should therefore be regarded as a hypothesis-generating observation rather than evidence of subtype-specific protection or maternal imprinting. Determining whether maternal immune history contributes to such differences will require adequately powered analyses stratified by maternal birth cohort and pre-vaccination RSV-A- and RSV-B-specific neutralizing antibody titers [21]. These efficacy data do not show that maternal vaccination transfers a subtype-biased imprint to the infant or alters the infant’s subsequent active memory response.

The passive-antibody environment created by maternal RSVpreF vaccination is striking in magnitude. In the 2026 phase 4 interim study of RSVpreF versus nirsevimab [104], maternal vaccination boosted maternal RSV-A neutralizing antibody titers 17.35-fold at delivery, with placental geometric mean transfer ratios above 1.3 for both subtypes. Infants born to vaccinated mothers had highly elevated neutralizing titers at 6 weeks and 3 months, with modest between-group differences in waning. Passive antibody thus dominates the infant serological environment during the first months of life. This has two immunological consequences. First, during the window of highest disease risk (0 to 6 months), the infant is protected by maternal antibodies whose epitope specificity is skewed toward site Ø and site V because the maternal vaccine is prefusion-stabilized [99,100]. Second, this dense passive antibody environment coincides precisely with the infant’s first natural exposure to RSV in most cases. Whether pre-existing maternal antibody biases or dampens the developing infant B-cell repertoire remains unresolved. Antibody-feedback mechanisms documented for SARS-CoV-2 and influenza [25,78,79] predict that high maternal titers directed at site Ø and site V could mask these epitopes during the infant’s first encounter, thereby shifting the infant’s active response toward alternative sites or delaying its onset. Ongoing follow-up of MATISSE infants at 24 months and the phase 4 kinetics study through 12 months will address this question directly [21,104].

### 5.2. Nirsevimab and Passive Immunization as Potential Modifiers of Subsequent Imprinting

Nirsevimab occupies a distinct position in the imprint-aware framework. It is a fully human IgG1 monoclonal antibody, engineered from D25 and directed against antigenic site Ø of the prefusion F glycoprotein [105], with the YTE Fc modification that extends serum half-life to approximately 63 to 73 days [105]. A single intramuscular dose provides therapeutic serum concentrations throughout an entire RSV season without eliciting an active B- or T-cell response in the recipient. Because nirsevimab provides passive protection without directly activating the recipient’s B- or T-cell responses, it does not itself establish active immunological memory. Whether it delays, redirects, or otherwise alters the imprint generated by subsequent natural RSV exposure remains unknown.

The efficacy of nirsevimab as passive prophylaxis is now unambiguous. In the pivotal MELODY phase 3 trial in healthy late-preterm and term infants, nirsevimab reduced medically attended RSV-associated lower respiratory tract illness by 74.5%; the estimated 62.1% reduction in RSV-associated hospitalization was not statistically significant (95% CI, −8.6 to 86.8; *p* = 0.07) [106]. In the pragmatic HARMONIE trial across France, Germany, and the United Kingdom, nirsevimab prevented 83.2% (95% CI 67.8 to 92.0) of hospitalizations for RSV-associated lower respiratory tract infection and 75.7% (95% CI 32.8 to 92.9) of very severe episodes defined by oxygen saturation below 90% and the need for supplemental oxygen [107]. Real-world evidence from 2023–2026 has consolidated these estimates across geographies. A systematic review and meta-analysis of 32 observational studies (27 pooled) from five countries found that nirsevimab was associated with lower odds of RSV-related hospital admission (odds ratio 0.17; 95% CI 0.12–0.23; I^2^ = 85.8%) and of intensive care unit admission (odds ratio 0.19; 95% CI 0.12–0.29; I^2^ = 55.6%) among infants aged 0–12 months, corresponding to approximately 83% and 81% effectiveness, respectively [108]. The Spanish NIRSE-GAL cohort reported 82.0% effectiveness against RSV hospitalization and 86.9% against severe RSV requiring oxygen supplementation [109]. In the United States, a multicenter case–control study at 27 pediatric hospitals across 24 states during the 2024–25 RSV season estimated nirsevimab effectiveness of 80% (95% CI, 70–86) against RSV-associated ICU admission and 83% (95% CI, 74–90) against acute respiratory failure. Effectiveness against ICU admission declined from 86% (95% CI, 77–92) at 7–59 days to 66% (95% CI, 47–79) at 60–183 days [110]. An earlier New Vaccine Surveillance Network estimate for 2023–24 showed 90% (95% CI, 75–96) effectiveness against RSV-associated hospitalization [111]. Passive prophylaxis therefore functions as intended: a high-affinity site Ø antibody delivered at a defined titer converts the first RSV season from a period of substantial morbidity into one of near-full protection for the majority of dosed infants.

The imprinting question begins where passive protection ends. Because nirsevimab does not stimulate the infant immune system, the emerging memory response to RSV must arise either from a breakthrough or subclinical infection during the covered season or from a first natural infection in the second year of life, when serum concentrations no longer neutralize the virus. Two lines of evidence address whether these deferred exposures generate a normal imprint. First, MELODY and its companion trial followed participants through a second RSV season without further prophylaxis. In that season, the incidence of medically attended RSV-associated lower respiratory tract illness was 1.8% with nirsevimab and 2.1% with placebo, and severity was comparable. Therefore, neither enhanced disease nor a deferral of the infection burden to the following year was observed [106,112]. Second, Wilkins and colleagues reported similar postfusion-F seroresponse rates in nirsevimab recipients (68–69%) and placebo recipients (63–70%), indicating that prophylaxis did not preclude an active response after RSV exposure [113]. Recent age-stratified humoral profiling by Nziza and colleagues [114] extended this observation to the population level: maternal RSV antibodies were not found to imprint or bias infant humoral immunity in ways detectable at 12 or 24 months, suggesting that passive antibody at physiologic concentrations does not overwrite the developing repertoire. These findings demonstrate preserved serological responsiveness after passive prophylaxis but do not provide direct evidence that the underlying memory B-cell repertoire remains unchanged. The available studies have not longitudinally compared clonal composition, somatic hypermutation, epitope specificity, or recall capacity between nirsevimab-exposed and unexposed children.

Nevertheless, whether a delayed first RSV infection produces a qualitatively different imprint than an infection at 2 to 6 months remains open. Mathematical models by Zheng and colleagues predict that, under a maternal immunization program, the average age of first RSV infection would increase from 14.3 to 19.2 months; extended-half-life monoclonal antibodies were predicted to have a similar directional effect, but the corresponding numerical ages were not reported [115]. The immunological consequences of this shift are not trivial. Neonatal and early-infant immune responses to RSV are characterized by suboptimal type I and type II interferon signaling, biased Th2 and Th17 polarization, and weaker germinal center output than adult responses [98]. A first infection at 18 to 24 months encounters a more mature immune system, potentially generating a broader and more durable memory repertoire, but also a system in which cross-priming by other respiratory viruses (particularly hMPV, parainfluenza, and endemic coronaviruses) may already have shaped the T-follicular helper landscape. Whether delayed first infection establishes protective cross-reactive memory, and whether any accompanying first-antigen bias is neutral or restrictive for subsequent RSV seasons, and whether it alters the site Ø versus site II and IV hierarchy of the elicited repertoire, has not been resolved by follow-up beyond 24 months.

Two operational implications follow. The first concerns the intervention’s durability. Nirsevimab provides protection during the immunologically vulnerable window without establishing an active memory record, so the population benefit is fully contingent on continued annual coverage of successive birth cohorts. Interrupting the program in one season would leave that cohort naive at older ages, when disease severity is lower but not negligible. The second concerns interpreting subsequent vaccine studies. Any future infant or toddler RSV vaccine trial conducted in nirsevimab-experienced cohorts will need to distinguish responses in children whose first RSV exposure occurred during passive coverage (potentially attenuated or subclinical) from those whose first exposure occurred after antibody waning (natural, symptomatic, and later in life). The imprint-modification hypothesis is testable through longitudinal serologic and cellular profiling of nirsevimab-recipient cohorts through the third and fourth years of life, and such studies are now feasible given the scale of national implementation programs [108,109,111]. Whether nirsevimab is best conceptualized as a bridge to natural imprinting, as a substitute imprint, or as an imprint modifier will depend on data that the ongoing 2026 surveillance cohorts are only beginning to generate.

Current evidence therefore supports the clinical effectiveness of passive RSV prevention and the preservation of subsequent serological responses, but not the modification of long-term immune imprinting. The RSV imprinting hypothesis remains unproven and should be tested in longitudinal birth cohorts that incorporate antigen-specific memory B-cell phenotyping, clonal B-cell receptor sequencing, epitope-resolved serology, and follow-up across multiple natural RSV exposures.

### 5.3. RSV-A Versus RSV-B and the Two-Subtype Imprint Question

RSV circulates as two antigenic subgroups, A and B, defined by sequence divergence in the attachment G glycoprotein and by surface epitope differences on the prefusion F trimer. G shares only 35% amino acid identity between subgroups, whereas F is more conserved and drives most cross-neutralizing activity in adult sera [116]. The two subgroups alternate as the dominant circulating lineage from season to season, with regional A-B-A-B or A-B-B rotation patterns shaped by strain-specific herd immunity [117,118]. This alternation is not accidental. Household transmission studies and prospective birth cohorts show that homotypic immunity, meaning protection against the same subgroup, is stronger and more durable than heterotypic immunity against the opposite subgroup. Molecular analyses of consecutive RSV infections in infancy indicate loss of strain-specific immunity within 7 to 9 months and loss of group-specific immunity within 2 to 4 months during a single epidemic season [119]. Over longer timescales, the subgroup encountered first appears to leave a measurable imprint on the neutralizing repertoire. In one report, adults recovering from natural RSV infection during a season dominated by RSV-B mounted stronger neutralizing responses against RSV-B, while T-cell polyfunctionality was higher against RSV-A F peptides, consistent with cross-reactive T-cell memory laid down by earlier RSV-A exposure in childhood [116]. The classical framing of RSV immunity as a simple failure of memory misses a more accurate picture: lifelong reinfection reflects an incompletely renewed, subgroup-biased memory pool whose original imprint remains detectable decades after primary infection.

The three licensed adult RSV vaccines were designed against this imprinted background. Arexvy, from GSK, is a monovalent recombinant prefusion F antigen from an RSV-A strain, adjuvanted with AS01E [120]. In the AReSVi-006 phase 3 trial in adults 60 years and older, a single dose showed 82.6% efficacy against RSV-associated lower respiratory tract disease in the first season, with sustained protection, including 62.9% cumulative efficacy (97.5% CI, 46.7–74.8) over three seasons and 48.0% efficacy (95% CI, 8.7–72.0) in the third season [121]. Abrysvo, from Pfizer, is a bivalent unadjuvanted prefusion F vaccine that includes stabilized F from both an RSV-A and an RSV-B lineage. In the RENOIR phase 3 trial in adults 60 years and older, Abrysvo showed 66.7% efficacy against RSV-associated lower respiratory tract illness with at least two symptoms and 85.7% efficacy against illness with at least three symptoms [122]. mRESVIA, from Moderna, encodes stabilized prefusion F from RSV-A on a lipid-nanoparticle-formulated modified mRNA platform [123]. In the ConquerRSV phase 3 trial, mRESVIA showed 83.7% efficacy against RSV-associated lower respiratory tract disease with at least two symptoms and 82.4% efficacy against illness with at least three symptoms, with protection observed against both RSV-A and RSV-B subtypes [123]. Two of the three licensed products, Arexvy and mRESVIA, therefore raise a monovalent RSV-A immunogen in a population whose infant imprint was formed against whichever subgroup happened to dominate in the year each recipient was born, roughly 60 to 90 years earlier [116,120,123]. Whether that ancient imprint constrains, guides, or is simply overwritten by the vaccine-elicited response remains an open empirical question with direct efficacy consequences.

The maternal MATISSE trial of bivalent RSVpreF provides a hypothesis-generating observation about possible subgroup-specific differences in protection transferred to infants. In the final analysis through 180 days after birth, vaccine efficacy against severe medically attended RSV-associated lower respiratory tract illness was 75.5% against RSV-B (12 versus 49 cases; 95% CI 53.3 to 88.1) and 52.9% against RSV-A (8 versus 17 cases; 95% CI −15.1 to 82.4). The RSV-A estimate was based on a small number of cases in the placebo group and was not statistically significant. Consequently, the apparent difference may reflect case availability and unequal statistical power and cannot establish biological heterogeneity in vaccine protection or a causal contribution of maternal imprinting. One testable, but currently unconfirmed, hypothesis is that maternal childhood exposure history may influence the bivalent immunogen’s relative boosting of anti-RSV-A and anti-RSV-B repertoires. Evaluating this possibility will require adequately powered analyses stratified by maternal birth cohort and pre-vaccination subtype-specific neutralizing antibody titers [4,21].

RSV is the first respiratory-virus vaccine program in which imprint-awareness has entered the design conversation from the beginning rather than as a post hoc correction. Structural biology defined the pre-F conformation and its dominant neutralizing epitope sites Ø and V before any product was licensed, and both were preserved in Arexvy, Abrysvo, mRESVIA [99,100]. This structural precision means that the first vaccine-induced imprint in each recipient, whether newborn, infant, or older adult, can in principle be directed at defined epitopes with known cross-subgroup breadth, rather than at the drifted G ectodomain or postfusion F which dominates natural infection. The pediatric analog matters as well. Suppose nirsevimab or clesrovimab covers the first RSV season, and the child’s first natural RSV exposure occurs in the second year of life against a mature immune system. The imprint laid down at that later encounter could, in principle, preferentially expand pre-F-directed and site-V-directed memory, in contrast to the G-dominated primary response seen in unprotected infants at 2 to 6 months [114,124]. The pediatric field is therefore approaching a natural experiment on a population scale. Are children whose first RSV encounter is deferred to 18 to 24 months and then met by a history of earlier nirsevimab exposure developing a broader, more cross-subgroup, more pre-F-focused memory pool than the cohorts imprinted at 2 to 6 months in the pre-nirsevimab era? Longitudinal serology and repertoire analysis in the CAUSAL-RSV and NIRSE-GAL cohorts should begin to answer this question over the next three to five seasons [109].

The broader conceptual point is that RSV has forced the vaccine field to accept, as influenza and SARS-CoV-2 did not initially, that imprinting is a design variable rather than a bug. Prefusion F stabilization, subgroup coverage, delivery timing, and passive-active sequencing are now weighed against their expected effects on the recipient’s future memory landscape [125]. Over the next decade, the three RSV products described above will generate the first prospective dataset in which imprint effects can be tracked from the moment the first neutralizing antibody appears, whether transferred passively as nirsevimab, elicited by maternal Abrysvo, or induced years later by mRESVIA or Arexvy in the same person as an older adult. The lessons from that dataset should shape the design of universal influenza vaccines, next-generation SARS-CoV-2 boosters, and any future respiratory-virus vaccine intended for imprinted populations.

## 6. When Cross-Reactive Memory May Help and When First-Antigen Bias May Restrict: A Hypothesis-Generating Conceptual Framework

The three viral systems reviewed above are typically treated as separate case studies of immune imprinting. That framing obscures a common underlying immunological process: priming a memory pool that then dominates subsequent responses, which manifests with different clinical signs. Group-matched childhood exposure is associated with protection against H5N1, whereas ancestral-spike bias can restrict early responses to Omicron [15]. We therefore use three qualitative dimensions to organize hypotheses about when cross-reactive memory may remain useful and when preferential first-antigen bias may restrict responses to a later antigen. SARS-CoV-2 imprinting on the ancestral spike constrained the initial neutralizing response to Omicron during 2021–2022 [19]. For RSV, the MATISSE subgroup estimates raise the hypothesis that maternal exposure history may influence the repertoire boosted by a bivalent maternal vaccine, but this remains unproven [21]. Because useful cross-reactive memory and preferential first-antigen bias may coexist, evaluating a new pathogen requires a framework that separately accounts for these two consequences of immune history.

In the qualitative cross-pathogen scheme used here, the three variables considered below are treated as factors that may influence two related but distinct outcomes: the utility of cross-reactive memory and the extent to which preferential first-antigen bias constrains responses to a later antigen. The first is the antigenic distance between the priming immunogen and the challenging strain. The second is the accessibility of subdominant epitopes on the updated immunogen, that is, whether conserved epitopes are exposed to naive B cells while memory recall is active. The third is the interval between prime and boost or between successive natural exposures, which may influence how much of the acute memory pool has contracted at re-exposure. Each of the three virus systems in this review illustrates a different combination. The dimensions are not combined in a fitted model, no validated weights or interaction rules are specified, and the scheme is not intended for ex ante prediction. The three virus systems serve as illustrative cases rather than an independent validation dataset.

The present three-variable framework is primarily limited to B-cell-mediated humoral imprinting and does not model the full contribution of T-cell memory. T-cell imprinting can independently modify protection and immunopathology through the specificity, functional polarization, tissue localization, and recall capacity of antigen-experienced CD4+ and CD8+ T cells. This limitation is particularly relevant to FI-RSV vaccine-enhanced disease, in which poorly protective antibody responses were accompanied by pathogenic, Th2-skewed CD4+ T-cell memory, with distinct CD4+ T-cell subsets contributing to different manifestations of enhanced disease [126]. Influenza provides a complementary example: early exposure history shapes the immunodominance and functional profile of influenza-specific CD4+ T-cell responses, including responses to conserved internal proteins such as nucleoprotein and matrix protein 1 [127]. Accordingly, hypotheses derived from the three qualitative dimensions address only the humoral component of imprinting and should not be interpreted as predictions of the complete immune outcome. Future expansion of the framework will require additional variables describing T-cell epitope conservation, HLA restriction, functional polarization, and tissue-resident memory.

### 6.1. Relationship to Recent Mathematical Models

Recent mathematical studies provide a quantitative complement to the qualitative framework used here. Yang et al. developed DynaVac, a model of antigen-specific B-cell dynamics that explicitly represents competition between memory and naive B-cell compartments and incorporates empirical cross-neutralization matrices, antigenic distance, vaccine dose, exposure interval, sequential exposure, and heterologous or multivalent vaccine composition. The model was parameterized using 29 murine SARS-CoV-2 vaccination regimens and tested against an independent murine dataset, with additional fits to macaque and human neutralization data. In a specified two-prime/two-boost simulation, it defines protection, pitfall, and breakthrough zones in which updated vaccination respectively amplifies, is suppressed by, or bypasses pre-existing immunity. DynaVac therefore shows that several variables considered qualitatively in the present review can be jointly parameterized within a pathogen-specific quantitative framework. These boundaries are model- and regimen-dependent rather than universal, and the model does not resolve epitope-specific clones, T-cell immunity, Fc-mediated functions, or protection against severe disease. However, its calibrated performance for SARS-CoV-2 regimens does not validate the present cross-pathogen scheme or provide transferable thresholds for influenza and RSV [128].

Chardès et al. addressed a related question using optimal decision theory. Their model examines the conditions under which recall of an existing repertoire or the recruitment and affinity maturation of a de novo response better balance future immune coverage against immediate immune and metabolic costs. The preferred response depends on antigenic divergence, repertoire diversity and cross-reactivity; the availability of de novo responders; and the costs of repertoire adaptation. This work provides a theoretical explanation for persistent recall when memory remains sufficiently useful, but it does not explicitly model vaccine dose, exposure interval, multivalent composition, or cellular competition for antigen and T-cell help [129].

Complementary pathogen-specific models extend this quantitative landscape. Urdy et al. developed a mechanistic multi-strain influenza model for older adults that represents strain-specific antibodies and adaptive effector and memory populations, links cross-reactivity to antigenic distance, and evaluates standard- versus high-dose vaccination across H1N1 and H3N2 seasons. It is informative for strain matching and dose effects, but it does not directly model childhood first-exposure bias [130]. A calibrated CD4+ T-cell model developed by Zhang et al. predicts that the weakest secondary response may occur at intermediate memory cross-reactivity, when rapid activation of cross-reactive memory induces regulatory signals that prematurely suppress the naive CD4+ T-cell response [131]. This general CD4+ T-cell model is not influenza-specific, but it provides a mechanistic example of T-cell imprinting and supports extending future frameworks beyond humoral memory.

Together, these studies clarify the framework’s position. DynaVac is the closest quantitative implementation of jointly acting exposure and vaccine-design variables, whereas the Chardès, influenza, and T-cell models formalize complementary aspects of repertoire optimization, strain-specific vaccine effectiveness, and T-cell memory-mediated regulation of secondary responses. The three-dimensional scheme used here is intended as a cross-pathogen organizational layer that generates testable hypotheses; predictive use would require pathogen-specific parameterization, explicit interaction rules, and independent validation [128,129,130,132].

### 6.2. Qualitative Dimensions and Illustrative Cross-Virus Mapping

Here, antigenic distance is treated as a qualitative constraint on the potential utility of memory recall. At relatively short distances, such as annual seasonal drift in influenza HA1 or minor drift within an Omicron subvariant clade, cross-reactive memory may retain sufficient affinity to recognize the later antigen [36]. At larger distances, outcomes remain context-dependent: memory directed toward conserved group-level epitopes may remain useful, as illustrated by HA-group-associated protection against H5N1 and H7N9, whereas recall directed toward poorly conserved or obsolete epitopes may restrict de novo responses, as reported for the ancestral-to-Omicron transition [19,74]. The conceptual scheme therefore does not treat long antigenic distance as intrinsically maladaptive or specify a universal transition.

For prospective applications, serum-based antigenic cartography provides the preferred primary measure of antigenic distance. Maps should be constructed from homologous and heterologous hemagglutination-inhibition titers for influenza or neutralization titers for SARS-CoV-2, with one antigenic unit corresponding to a twofold reduction in cross-reactive titer [133,134]. In this conceptual scheme, “short,” “intermediate,” and “long” are descriptive, pathogen- and assay-dependent categories rather than universal quantitative cut-offs. The framework does not assign universal thresholds, weights, or interaction rules, and it has not been jointly fitted or independently validated. These descriptive categories organize context-dependent observations and hypotheses. No category by itself maps to an outcome or supports ex ante prediction. These cut-offs are heuristic rather than universal biological boundaries and require prospective validation. Deep mutational scanning provides a complementary epitope-level measure by identifying mutations that escape antibodies elicited by the imprinting antigen; because escape scores depend on the tested sera and assay normalization, they should not be converted directly into universal antigenic-distance thresholds.

Subdominant epitope accessibility is treated here as a qualitative factor that may influence whether a recall response broadens. Chimeric hemagglutinin vaccines pairing conserved stalks with divergent heads, mosaic RBD nanoparticles displaying sarbecovirus receptor-binding domains, and hyperglycosylation strategies that mask the immunodominant head all operate on the same principle. Each dampens memory recall to the dominant epitope and forces naive B-cell activation against subdominant conserved sites [135]. The RSV prefusion F antigen accomplishes something analogous by presenting site Ø and site V in a stable conformation that is otherwise transient on the wild-type virus [99,100]. For purposes of this conceptual scheme, the relevant dimension is the relative accessibility of immunodominant and subdominant epitopes at the time of B-cell selection; no universally calibrated ratio is assumed.

We treat the interval between exposures as a qualitative, context-dependent factor that may influence the balance between memory recall and de novo priming. Study-specific intervals should not be interpreted as universal cut-offs. Reports that a bivalent BA.5 booster given three months after a WT booster produced deep imprinting, whereas repeated Omicron exposures across a full year progressively broadened the response, are consistent with an interval-related hypothesis [19,74]. Maternal Abrysvo exposure followed by natural RSV infection likewise motivates the unvalidated hypothesis of a broader, more pre-F-focused repertoire; longitudinal testing is required [21].

**Figure 3:**
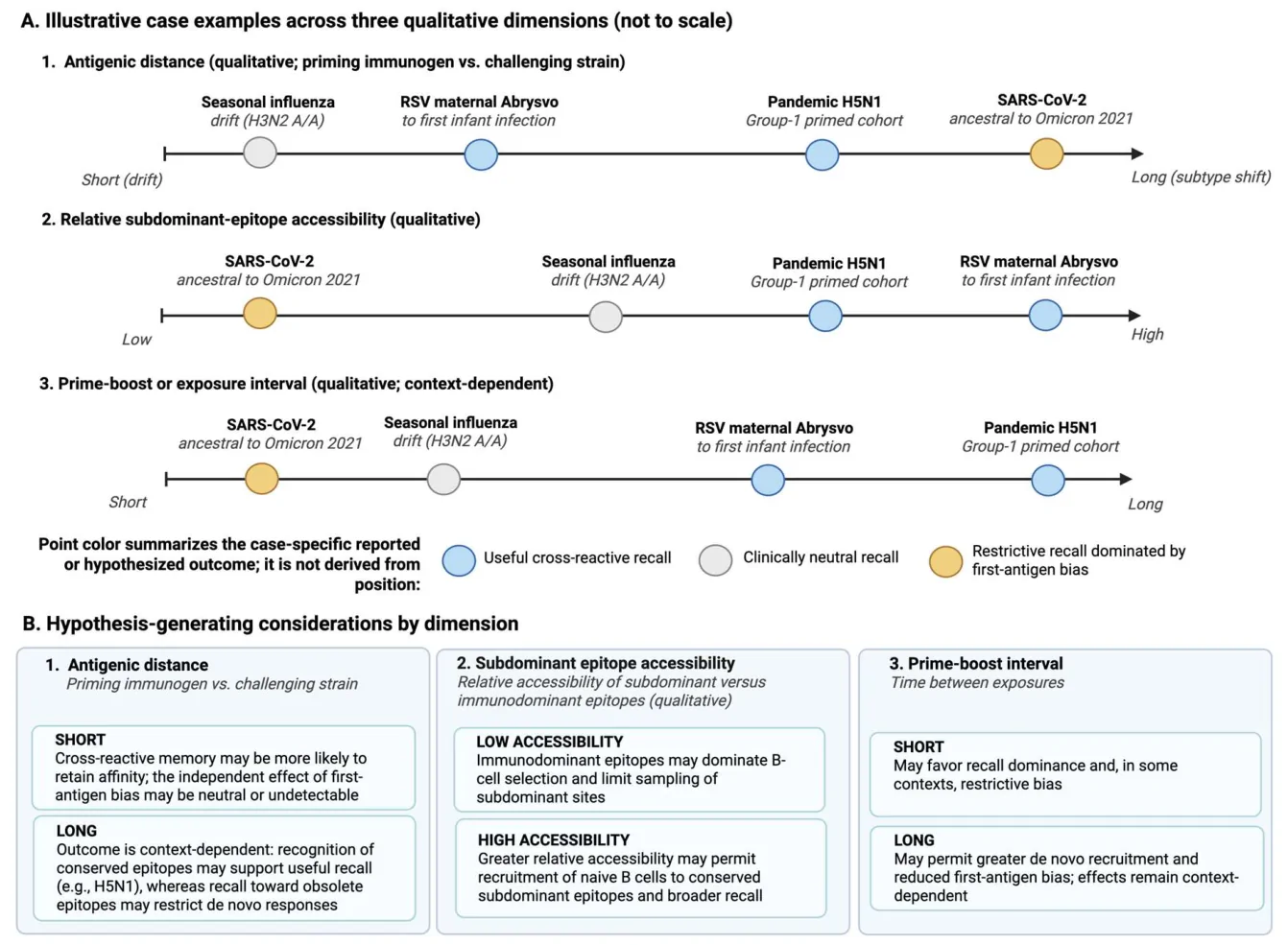
Hypothesis-generating conceptual scheme for cross-reactive memory utility and first-antigen bias. (**A**) Four examples from this section are placed qualitatively, and not to scale, along three descriptive dimensions: antigenic distance between the priming immunogen and the challenging strain, relative subdominant-epitope accessibility on the updated immunogen, and the prime–boost or exposure interval. Point color summarizes the case-specific outcome reported in the cited evidence or, for maternal RSV vaccination, explicitly hypothesized; it is not calculated from point position. Blue indicates useful cross-reactive recall, gray indicates clinically neutral recall, and orange indicates restrictive recall dominated by first-antigen bias. These categories describe net recall outcomes and do not imply that preferential first-antigen bias is beneficial. H5N1 is intentionally placed at a qualitatively long antigenic distance with useful cross-reactive recall, illustrating that long distance is not intrinsically maladaptive when conserved group-level epitopes remain recognized. (**B**) Qualitative, hypothesis-generating considerations for each dimension. The statements indicate possbold removedible context-dependent tendencies and are not decision rules, fitted relationships, or independently validated predictions. No weighting or interaction rule is specified, and the dimensions should not be used to predict outcomes ex ante. These qualitative outcome categories should not be equated with the quantitatively parameterized protection, pitfall, and breakthrough zones defined in DynaVac [128]. Prospective datasets are required to test the hypotheses suggested by the scheme. Created in BioRender. Szuster-Ciesielska, A. https://BioRender.com/gqccb13, accessed on 20 August 2026.

Mapping the three virus systems onto these qualitative dimensions illustrates how existing observations and untested hypotheses can be organized; it does not constitute prospective prediction. Seasonal influenza drift occurs over short antigenic distances, with intermediate epitope accessibility and short intervals; cross-reactive recall is generally useful or clinically neutral, while the independent effect of first-antigen bias is difficult to isolate. In the H5N1 example, childhood group-1 HA exposure is associated with protective cross-reactive memory despite the large antigenic distance. These data do not establish that a preferential bias toward the first HA independently contributes to protection [15]. SARS-CoV-2 ancestral to Omicron occurs at a long distance, with initially poor subdominant epitope accessibility and short vaccine intervals, and imprinting was initially restrictive but was substantially mitigated after two or more variant exposures at longer intervals [19]. For RSV maternal vaccination, the framework generates a testable hypothesis that high pre-F epitope accessibility and the interval to first natural exposure may favor the establishment of useful pre-F-directed cross-reactive memory, but longitudinal infant data are not yet available. Figure 3 illustrates the qualitative placement of these examples across the three dimensions.

This conceptual scheme is valuable for organization and hypothesis generation. Antigenic cartography and epitope-level assays may help characterize antigenic distance [133,134,136]; competition-binding studies may inform relative epitope accessibility [135]; and measurements of plasmablast decay and germinal-center persistence may inform the exposure-interval dimension [78]. These approaches may support future operationalization of the individual dimensions. In the present manuscript, however, the dimensions are qualitative, are not combined using fitted parameters or an interaction rule, and have not been evaluated against an independent validation dataset. They should therefore be used to formulate testable hypotheses, not to estimate the expected utility or probability of an imprinting outcome for a new virus or vaccine design.

## 7. Imprint-Aware Vaccine Design: Strategies and Open Questions

**Figure 4:**
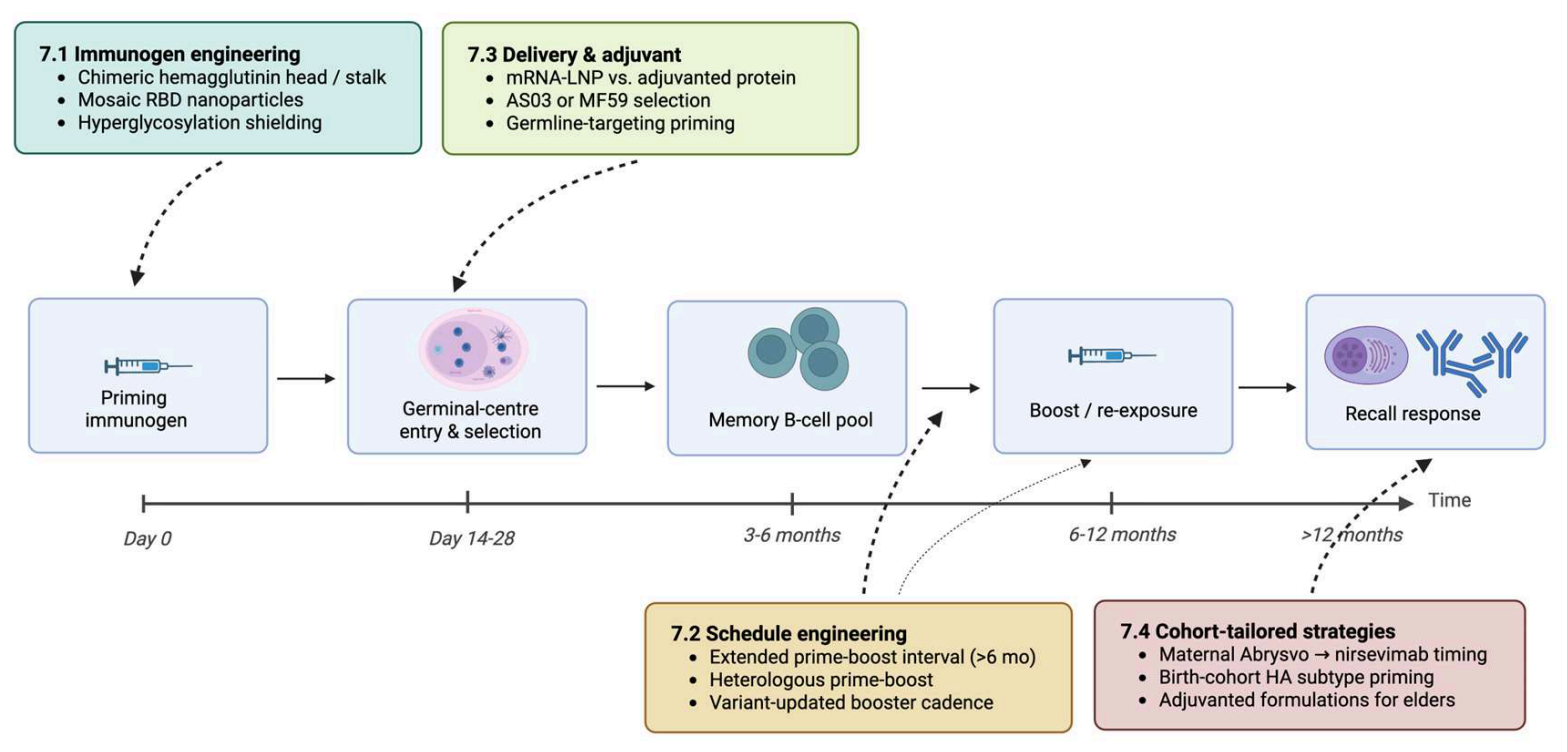
Imprint-aware vaccine design levers mapped onto the imprint life cycle. Dashed arrows indicate the mechanistic step each lever family targets. Bullets list representative strategies; Table 3 details the evidence tier. The central schematic shows the sequence from priming immunogen through germinal center entry and selection, memory B-cell pool formation, boost or re-exposure, and recall response. Four families of design levers act at distinct points in this sequence. Immunogen engineering modifies the priming antigen using chimeric hemagglutinin constructs, mosaic receptor-binding domain nanoparticles, or hyperglycosylation of immunodominant surfaces. Delivery platform and adjuvant selection shape germinal center entry and selection by choosing messenger RNA lipid nanoparticles versus adjuvanted protein, using AS03 or MF59, and employing germline-targeting priming. Schedule engineering extends the prime–boost interval, employs heterologous prime–boost combinations, and sets the cadence of variant-updated boosters to allow memory contraction before re-exposure. Cohort-tailored strategies match the recipient’s imprint history to the intervention, exemplified by maternal Abrysvo followed by nirsevimab in infants, birth-cohort hemagglutinin subtype priming in adults, and adjuvanted formulations for older adults with contracted memory pools. These intervention families are discussed in relation to the three qualitative dimensions introduced earlier in this review; this organization does not constitute a predictive algorithm. The time axis is approximate; intervals depend on the pathogen and cohort. The intervention categories are positioned according to the stage of the immune-response timeline at which they primarily operate. Created in BioRender. Szuster-Ciesielska, A. https://BioRender.com/nnc3lw1 accessed on 22 August 2026.

The combined mechanistic and virus-specific evidence raises a practical question: can vaccines preserve or broaden useful cross-reactive memory while minimizing restrictive bias toward the first antigen? Four principal design levers are considered: immunogen structure, delivery platform and adjuvant selection, booster timing and composition, and the passive-antibody environment in which active immunization occurs. Table 3 summarizes candidate strategies and their current stage of clinical or preclinical development. Figure 4 links each design lever to the mechanistic step it targets.

The section is deliberately honest about what has been demonstrated in humans and what remains hypothesis-generating. Chimeric hemagglutinin (HA) constructs have completed Phase 1 and Phase 2 trials, with immunogenicity readouts against conserved stalk epitopes. Mosaic receptor-binding domain (RBD) nanoparticles have shown cross-clade protection in nonhuman primates but have not yet entered efficacy trials in humans. Prefusion F immunogens for respiratory syncytial virus (RSV) are licensed and have generated the first large observational datasets on infant imprinting in the nirsevimab era. Hyperglycosylation, epitope resurfacing, and germline targeting remain preclinical for most pathogens. Distinguishing these evidentiary tiers is essential. Overinterpreting animal data as translational proof has repeatedly delayed rational design in this field.

### 7.1. Translational Status of Engineered Immunogens

Three immunogen-engineering approaches have advanced the furthest: chimeric hemagglutinin constructs, mosaic RBD nanoparticles, and hyperglycosylated antigens [41,135,136]. Their shared objective is to reduce the dominance of variable epitopes while preserving or enhancing the presentation of conserved sites. Chimeric hemagglutinin is the most clinically advanced approach. Following preclinical proof of concept, Phase 1 and Phase 1/2 studies demonstrated that sequential chimeric hemagglutinin vaccination induces durable, broadly cross-reactive stalk antibodies, particularly when combined with AS03 [42,43,137]. Stalk-specific CD4+ T-cell responses have also been observed. However, clinical efficacy against influenza infection or severe disease has not yet been demonstrated [138].

Mosaic RBD nanoparticles and hyperglycosylated immunogens remain preclinical. Mosaic-8 particles elicited cross-reactive neutralization and heterologous protection in mice and nonhuman primates, including animals previously vaccinated against SARS-CoV-2 [139,140]. Hyperglycosylated influenza and coronavirus antigens redirected responses away from variable immunodominant surfaces and toward conserved subdominant epitopes [141]. Their further development will require confirmation that glycan shielding does not compromise antigen folding or the accessibility of protective epitopes.

### 7.2. Schedule Engineering: Intervals and Heterologous Prime–Boost

If immunogen engineering acts on the antigen, schedule engineering acts on the immune system’s temporal behavior. Two variables dominate: the interval between doses and the identities of successive priming and boosting agents. Both influence which memory B cells re-enter germinal centers, how much time naive B cells have to compete for antigens, and how strongly prior exposures imprint and constrain the response to a new variant.

Observational evidence on the relationship between dosing interval and imprint plasticity comes from Wrynla and colleagues, who compared responses to the monovalent XBB.1.5 booster in participants who had received the intervening bivalent Wuhan-Hu-1/BA.4-5 vaccine with responses in those who had skipped it. Bivalent non-recipients had a longer mean interval since their previous dose, approximately 707 versus 361 days, but had also received one fewer vaccine exposure. They showed greater boosting of neutralizing antibodies against XBB.1.5 (18.4-fold versus 6.2-fold), EG.5.1 (30.9-fold versus 7.0-fold), and JN.1 (9.2-fold versus 3.7-fold), with comparable breadth. Both groups back-boosted titres against the ancestral WA1 spike, confirming that imprinting persisted under both exposure histories. However, interval length, bivalent-booster receipt, and the total number and antigenic composition of previous exposures were highly collinear. Consequently, the observed differences cannot be attributed independently to the longer interval, omission of the bivalent dose, lower cumulative exposure number, or interactions among these factors, and multivariable adjustment cannot fully resolve this structural confounding. The findings therefore demonstrate an association between exposure history and the response to an updated booster but do not provide a causal estimate of the independent effect of interval. Although the direction of the association is consistent with studies of extended intervals in the ancestral vaccine series, extrapolation to variant-updated boosting remains provisional [142]. Randomized trials that vary the interval while holding the number and antigenic composition of exposures constant, or factorial trials that manipulate these variables independently, are required to quantify their separate contributions [91].

Heterologous prime–boost is the second lever. The rationale is mechanistic. Successive doses on the same platform recall the same memory B cell pool via the same antigen-presentation pathway. Switching platforms alters the innate signals delivered during boosting (differences in Toll-like receptor engagement, type I interferon induction, and antigen persistence) and can broaden the epitope repertoire that dominates the recall response. In the Com-COV randomized trial, Liu and colleagues showed that a ChAdOx1 nCoV-19 prime followed by a BNT162b2 boost at a 4-week interval induced anti-spike IgG geometric mean concentrations of 12,906 ELU/mL, roughly 9.2-fold higher than homologous ChAdOx1/ChAdOx1 (1392 ELU/mL) [143]. Com-COV2, led by Stuart and colleagues, extended these findings to mRNA-1273 and Matrix-M-adjuvanted NVX-CoV2373 as boost agents and demonstrated non-inferiority or superiority of most heterologous schedules over homologous ChAd/ChAd [144]. Pozzetto and colleagues subsequently reported that heterologous ChAdOx1/BNT162b2 in a real-world cohort of 13,121 healthcare workers conferred better protection against symptomatic SARS-CoV-2 infection than homologous BNT162b2/BNT162b2, and correlated the improved outcome with a higher frequency of switched and activated RBD-specific memory B cells [145]. Immunologically, this suggests that the heterologous boost recruits a broader, less imprint-anchored B cell population, consistent with the general principle that changing the delivery modality partially uncouples the recall response from the initial imprint.

Delayed boosting is the third lever and mechanistically distinct from simply lengthening the prime–boost interval. Mesin and colleagues showed in a mouse model that memory B cells rarely re-enter secondary germinal centers, and that these centers are seeded primarily by naive B cells, regardless of whether boosting occurs one or three months after priming [27]. This suggests that a delayed boost does not so much erase memory as give naive B cells a competitive window to enter new germinal centers and mature against the current variant. In humans, several observational cohorts have evaluated deliberately extending the interval between doses to allow memory contraction as a plausible imprint-mitigation strategy. Tauzin and colleagues reported that a 16-week interval between the first two BNT162b2 doses produced more robust neutralizing responses against the ancestral virus and variants of concern in naive individuals than a standard 3- to 4-week schedule [146]. Parry and colleagues showed that extending the interval from 3 to 12 weeks increased peak spike-specific antibody titers by 3.5-fold in adults older than 80 years [147]. However, the available evidence is observational and confounded by circulating variant composition at the time of vaccination. Interval extension alone is also insufficient to overcome established imprinting. Yisimayi and colleagues found in a mouse model that a 3-month or 6-month interval between ancestral priming and an Omicron booster only marginally increased neutralization against XBB relative to a 1-month interval and that a second Omicron booster given after a 3-month interval was required for meaningful mitigation [19]. No randomized trial has yet tested a schedule specifically designed to permit memory contraction in an imprinted population against a variant-adapted immunogen. Schedule engineering is therefore best viewed as a partial, empirically supported lever, useful in combination with immunogen or platform changes rather than as a stand-alone solution.

### 7.3. Adjuvant Selection and Germinal-Center Modulation

Adjuvants are the fourth lever and mechanistically the one that most directly shapes germinal-center dynamics. For imprint-aware design, the relevant question is not whether an adjuvant increases antibody titer, but whether it prolongs germinal-center activity long enough to allow selection of naive B cells against novel epitopes and whether it broadens the recall response beyond epitopes shared with the imprinting antigen. Emulsion adjuvants such as MF59 and AS03, and the liposome-based Adjuvant System AS01, all engage innate pathways that support these outcomes, but through distinct mechanisms and with different consequences for imprint modulation. Pulendran and colleagues have summarized the current understanding of these mechanisms in a comprehensive review, emphasizing that adjuvants shape not only the magnitude but also the quality, breadth, and durability of the antibody response through germinal-center induction, follicular helper T cell activation, and long-lived plasma cell generation [148].

MF59, an oil-in-water squalene emulsion, promotes germinal-center reactions by inducing follicular helper T cells (Tfh). Mastelic-Gavillet and colleagues showed in a murine model that MF59 markedly increases Tfh cell frequencies in the draining lymph node and directly enlarges the germinal-center B cell compartment. This effect is preserved in three-week-old infant mice but not in one-week-old neonates [149]. In humans, MF59-adjuvanted seasonal influenza vaccines elicit broader homosubtypic and heterosubtypic antibody responses than non-adjuvanted comparators, particularly in immunologically naive children and in older adults with narrowed baseline repertoires. In a randomized field trial of 4707 children aged 6 to 72 months, Vesikari and colleagues showed that the MF59-adjuvanted trivalent influenza vaccine had 86% (95% CI, 74–93) efficacy against all influenza strains compared with 43% (95% CI, 15–61) for the non-adjuvanted comparator, with efficacy of 79% in children aged 6 to <36 months and 92% in those aged 36 to <72 months [150]. Khurana and colleagues, in a phage-display epitope mapping study of paired sera from adults and children, showed that MF59 selectively expanded the antibody repertoire against the HA1 globular head and increased antibody affinity by more than tenfold, as inferred from surface plasmon resonance off-rates [151]. Both effects were most pronounced in influenza-naive individuals, consistent with preferential action on naive B cells. Mechanistically, the breadth is attributable to two coincident processes. First, the adjuvant increases activation of naive B cells against novel head epitopes, thereby diluting the recall-dominant fraction of the response. Second, it re-engages preexisting memory B cells in ongoing germinal centers, where further affinity maturation refines their specificity. O’Hagan and colleagues have summarized this bimodal mechanism for emulsion adjuvants, arguing that MF59 and AS03 recruit naive B cells against novel epitopes while simultaneously re-engaging preexisting memory B cells for further affinity maturation, and that the balance of the two processes shapes the diversity of the resulting repertoire [152]. This bimodal recruitment provides a mechanistic route by which an adjuvant can, in principle, mitigate imprinting by expanding the fraction of the response that is not anchored to the initial antigen.

AS03, a squalene emulsion formulated with alpha-tocopherol, produces a more pronounced innate signature than MF59 and correlates with greater breadth in head-to-head comparisons. Goll and colleagues compared AS03-adjuvanted and MF59-adjuvanted H5N1 vaccines in humans and showed that at day 42 the AS03 arm elicited more than twice as many significant IgG responses across a panel of hemagglutinin antigens and broader coverage of non-H5 HA subtypes than the MF59 arm [153]. AS03 also produces a qualitatively distinct memory B cell response. Grigoryan and colleagues studied the plant-based CoVLP SARS-CoV-2 vaccine given with or without AS03 in a longitudinal design [154]. AS03 not only increased the magnitude and persistence of spike-specific memory B cells but also drove progressive accumulation of somatic hypermutations over six months, with a rising fraction of clones encoding broadly neutralizing antibodies against Omicron variants and SARS-CoV. The clonal analysis indicated that AS03 acted at two levels: it enhanced selection of memory B cells with broad specificity and prolonged the germinal-center window during which affinity maturation could refine that breadth.

AS01, a liposome-based system combining monophosphoryl lipid A and the saponin QS-21, operates through a distinct innate axis. Roman and colleagues have described how MPL and QS-21 synergize to induce a rapid interferon-gamma burst in the draining lymph node, which in turn drives Tfh cell differentiation, sustained germinal-center reactions, and antibody responses of optimized functional quality [155]. Coccia and colleagues established this mechanistic model, showing in mice and confirming in macaques and human recipients of the RTS,S malaria vaccine that within hours of injection, AS01 induces MPL- and QS-21-dependent IFN-gamma release from resident NK and CD8 T cells in the draining lymph node. They further showed that this early IFN-gamma is required for downstream dendritic cell activation and Th1 differentiation, and that macrophages, IL-12, and IL-18 coordinate the response [156]. AS01 is used in the herpes zoster and RSV vaccines for older adults, populations in whom baseline immunological fitness constrains the response to non-adjuvanted formulations. Lal and colleagues, in the ZOE-50 trial of the HZ/su subunit vaccine adjuvanted with AS01B, reported 97.2% efficacy against herpes zoster in adults aged 50 years and older, with efficacy preserved across the 50–59, 60–69, and >=70 age strata [157]. Papi and colleagues, in the AReSVi-006 trial of the RSVPreF3 OA vaccine adjuvanted with AS01E, reported 82.6% efficacy against RSV-related lower respiratory tract disease and 94.1% against severe RSV-related lower respiratory tract disease in adults aged 60 years and older during one RSV season [120]. Both trials show that AS01 can elicit protective responses in immunosenescent hosts, which is directly relevant to imprint-aware design in older adults, where the imprinting antigen was often encountered decades earlier and recall bias toward that antigen is strongest.

The most direct evidence that adjuvants can modulate imprinting at the germinal-center level in a vaccine platform designed for breadth comes from studies of chimeric hemagglutinin vaccines. The rationale of this platform, described by Krammer and Palese, is to break the immunodominance of the HA globular head, where imprinting occurs, by sequential exposure to chimeric constructs that share a conserved stalk but present divergent, immunologically novel head domains from avian subtypes, thereby refocusing the recall response toward the subdominant conserved stalk [158]. Nachbagauer and colleagues, in the first phase I clinical trial of this concept in healthy adults, showed that a prime–boost regimen using a live-attenuated chimeric HA followed by an AS03-adjuvanted inactivated chimeric HA induced broad, long-lasting, cross-reactive antibodies targeting the H1 stalk domain [43]. The AS03-adjuvanted arms produced substantially higher and more durable anti-stalk responses than non-adjuvanted comparators. Akhtar and colleagues extended this line to rhesus macaques immunized with sequential chimeric HA constructs sharing a conserved H1 stalk but presenting divergent head domains, with or without AS03 [45]. AS03 significantly increased the frequency of chimeric HA-specific germinal-center B cells at week three after the second dose, drove the generation of long-lived stalk-specific plasma cells in bone marrow that persisted for up to two years, and increased serum antibody durability. Together, the human and nonhuman primate evidence illustrates how adjuvant selection can act synergistically with immunogen engineering to broaden cross-reactive memory while mitigating restrictive first-antigen bias.

The trade-off is that adjuvants that prolong germinal-center activity and broaden memory recall enable broader sampling of novel epitopes only under specific conditions. Broadening is favored when the boosting antigen carries new epitopes not represented in the imprinted repertoire, as in a variant-updated immunogen or a mosaic construct that displays conserved but subdominant epitopes alongside heterologous variable ones, and when the interval between doses permits memory contraction. Cohen and colleagues demonstrated this principle with mosaic-8 receptor-binding domain nanoparticles co-displaying SARS-CoV-2 and seven animal sarbecovirus RBDs, which elicited broader neutralization of heterologous sarbecoviruses and protected against both SARS-CoV-2 and SARS-CoV challenge, whereas homotypic SARS-CoV-2 nanoparticles protected only against the matched strain [140]. When the boosting antigen is identical to the priming antigen, the same adjuvant effect that would recruit naive B cells against novel epitopes instead amplifies the recall of imprint-anchored clones, deepening rather than broadening the response. Schaefer-Babajew and colleagues mechanistically showed that pre-existing antibodies elicited by the priming SARS-CoV-2 mRNA dose feedback-regulate the recall germinal-center response through epitope masking on follicular dendritic cells, restricting naive B cells’ access to immunodominant epitopes and thereby constraining broadening under a homologous boost [78]. This mechanistic basis means adjuvant selection cannot be evaluated in isolation from antigenic design and schedule. The three levers act together, and the empirical evidence supports viewing adjuvant selection as a modulator whose direction of effect depends on the antigenic context in which it is deployed.

### 7.4. Cohort-Tailored Strategies

The three levers of immunogen, schedule, and adjuvant selection do not act uniformly across populations. Immune imprinting is, by definition, age-dependent because the identity and strength of the imprinting antigen are set by the first encounter, which occurs at a different point in life for every birth cohort [15]. This has direct operational consequences. A vaccine strategy optimal for a fifty-year-old with a deeply anchored H1N1 or ancestral-strain imprint is not optimal for an infant who has never encountered the pathogen, and a strategy optimal for a naive infant will not correct an entrenched imprint in an older adult. Cohort-tailored strategies formalize this reasoning and align antigen design, platform, and delivery route with the target population’s imprint status.

#### 7.4.1. Infants and Young Children: Establishing Broad Memory While Minimizing Future Bias

For infants and young children, the priority is to establish broad, durable immune memory while minimizing the risk of restrictive first-antigen bias in subsequent exposures. This can be achieved either through direct active immunization with a cleaner, structurally defined antigen or through passive strategies that bridge the vulnerability window without establishing a competing imprint. The pediatric RSV field illustrates both approaches. Nirsevimab, a long-acting monoclonal antibody targeting the prefusion F conformation, protects infants during their first RSV season without inducing a B-cell memory response. Drysdale and colleagues, in the HARMONIE phase 3b trial, reported 83.2% efficacy against RSV-associated lower respiratory tract infection requiring hospitalization and 75.7% efficacy against very severe RSV lower respiratory tract infection over the full first season [107]. The complementary maternal vaccination strategy uses RSVpreF given at 24 to 36 weeks of gestation to transfer preformed prefusion-specific IgG across the placenta. Kampmann and colleagues, in the MATISSE trial, reported 81.8% efficacy against medically attended severe RSV lower respiratory tract illness within 90 days after birth and 69.4% through 180 days [4]. Both approaches provide passive protection through antibodies targeting prefusion F without establishing active immune memory in the infant. The F conformation encountered during initial active priming could influence responses to subsequent childhood RSV exposures. However, whether this creates a durable bias toward the initially encountered antigen remains unknown; testing this imprinting hypothesis requires longitudinal follow-up. The influenza field is moving in the same direction: pediatric immunization schedules that expose children to seasonally updated inactivated or live-attenuated vaccines rather than to circulating natural infection shift the imprint from a randomly encountered strain toward the vaccine strain, which is more likely to be a broadly relevant contemporary antigen [159].

#### 7.4.2. Adolescents and Young Adults: Broadening Memory While Imprinting Remains Plastic

For adolescents and young adults who have already established an imprint but retain broad germinal-center plasticity, the priority is to broaden cross-reactive memory while reducing restrictive first-antigen bias. This population is where mosaic constructs, chimeric hemagglutinin regimens, and heterologous variant-updated boosters have the greatest expected value [57,160]. Germinal-center reactivity is preserved, epitope masking by a pre-existing antibody is intermediate rather than saturating, and the interval since first exposure is short enough that the imprint has not yet consolidated into a fixed serological ceiling. Turner and colleagues, using fine-needle aspiration of draining axillary lymph nodes in healthy adults after BNT162b2 mRNA vaccination, demonstrated that spike-specific germinal-center B cells and plasmablasts persisted at high frequencies for at least twelve weeks after the booster. Spike-binding germinal-center B cells persisted at or near peak frequency for at least 15 weeks after the first immunization in 8 of 10 participants [160], and remained detectable at week 29 in 10 of 15 participants (approximately two-thirds), providing direct evidence of preserved and prolonged germinal-center capacity in the young-adult cohort [161]. The pandemic influenza field has explicitly designed universal vaccine candidates around this cohort. In the first-phase I chimeric hemagglutinin trial, Nachbagauer and colleagues enrolled adults aged 18 to 39 years, where the response to a live-attenuated chimeric HA prime followed by an AS03-adjuvanted inactivated chimeric HA boost could be measured cleanly against the recent H1 imprint. The adjuvanted arms induced broad and long-lasting cross-reactive antibodies against the conserved H1 stalk domain [43]. Cohen and colleagues extended the logic of heterologous immunogen presentation with mosaic 8 receptor-binding domain nanoparticles co-displaying SARS-CoV-2 and seven animal sarbecovirus RBDs, which elicited broader heterologous neutralization and protected against both matched and mismatched sarbecovirus challenges relative to homotypic SARS-CoV-2 nanoparticles [140]. The same logic applies to variant-updated SARS-CoV-2 boosters in adults. Wang and colleagues showed that an XBB. 1.5 monovalent booster in previously uninfected individuals elicited 13.3-fold to 27.6-fold increases in serum neutralizing antibodies against XBB.1.5, EG.5.1, HV.1, HK.3, JD.1.1, and the emerging JN.1 variant, indicating a measurable de novo response even when the ancestral imprint was present [162]. Wang and colleagues further reported that a KP.2 monovalent booster increased serum neutralizing titers 6.1-fold to 7.8-fold against JN.1 lineage subvariants, including KP.2, KP.3, KP.3.1, KP.3.1.1, and XEC [163]. The back-boost against ancestral D614G and BA.5 was modest at 1.6-fold to 3.3-fold, a pattern that the authors interpreted as partial mitigation of the imprinting problem observed with the earlier bivalent BA.5 formulation.

#### 7.4.3. Older Adults: Compensating for a Consolidated Imprint

For older adults with a deep, decades-old imprint and immunosenescence-limited germinal-center capacity, broadening the response is difficult, and the priority shifts to compensating for the imprint. Adjuvanted platforms that force germinal-center activity in compartments that would otherwise not sustain it, such as AS01-adjuvanted subunit vaccines, are practical tools for this cohort. Both the ZOE-50 herpes zoster program and the AReSVi-006 RSV program deliberately target adults aged 50 years and older (ZOE-50) and 60 years and older (AReSVi-006), and trial results demonstrate that a properly adjuvanted subunit vaccine can elicit protective efficacy in a population where non-adjuvanted formulations previously failed [120,156,164,165]. In ZOE-50, Lal and colleagues reported 97.2% efficacy against herpes zoster, and in AReSVi-006, Papi and colleagues reported 82.6% efficacy against RSV lower respiratory tract disease and 94.1% against severe disease [120,157]. High-dose and MF59-adjuvanted influenza vaccines follow the same cohort logic and the Advisory Committee on Immunization Practices recommends them preferentially for adults aged 65 years and older. The December 2024 MMWR update further formalized this by recommending a second dose of the 2024–2025 COVID-19 vaccine for all adults aged 65 years and older six months (minimum two months) after the first 2024–2025 dose [166].

#### 7.4.4. Operational and Ethical Dimensions of Cohort-Tailored Programs

Cohort-tailored vaccination programs raise ethical and operational questions distinct from those of a uniform program. The operational side is well established. Age-stratified recommendations require robust birth-year records, functional immunization registries, and delivery infrastructure capable of identifying eligible birth cohorts and routing them to the appropriate product, which is more demanding than a single universal schedule. Groom and colleagues, in a systematic review of Immunization Information Systems, demonstrated that functional population-based registries measurably improve age-appropriate coverage and enable the reminder, recall, and eligibility-verification workflows that cohort-specific programs depend on [167]. The United Kingdom Joint Committee on Vaccination and Immunisation RSV strategy recommends maternal RSVpreF vaccination for pregnant persons and a separate Abrysvo (RSVpreF, Pfizer) program for adults aged 75 to 79 years [168]. Such programs illustrate that this level of stratification is feasible in a well-resourced national program but demands consistent messaging to distinguish the two products and their target populations. The ethical side is more delicate. Preferentially offering a specific vaccine product to one cohort implicitly implies that other products, or no product, are appropriate for others, and this differential must be justified by evidence and communicated transparently to avoid the perception of unequal access. When the cohort-specific product is more expensive, as adjuvanted or high-dose formulations typically are, cost-effectiveness analyses stratified by age become necessary to sustain the argument. Chit and colleagues, in a cost-effectiveness analysis of high-dose trivalent inactivated influenza vaccine versus standard-dose vaccine in adults aged 65 years and older, showed that the high-dose vaccine was cost-saving relative to the standard dose, dominating it in the cost-utility analysis with a 93% probability of being cost-saving [169]. Equity considerations require attention to whether the recommended product is actually available in all settings where the target cohort lives. The ethical framework articulated by Emanuel and colleagues in the Fair Priority Model, which grounds differential allocation in benefit maximization, priority to the worst off, equal moral concern, and avoidance of discrimination, provides the normative scaffolding for defending cohort-specific recommendations against the charge of unequal access [170].

The public health implications for pandemic preparedness follow directly. When a novel respiratory pathogen emerges, the age structure of the exposed population determines the optimal early vaccine strategy, and imprint mapping should be a routine early-response activity. If the emerging pathogen shares antigenic features with a virus that has circulated in defined birth cohorts, as with the 2009 H1N1 pandemic, where older adults had cross-reactive memory associated with early exposure to 1918-lineage viruses, those cohorts can be deprioritized for the initial vaccine deployment, and doses can be directed to naive cohorts who are at highest risk [15,49]. Conversely, when the emerging pathogen is antigenically novel to the entire population, as SARS-CoV-2 was in 2020, the entire population is functionally imprint-naive at first exposure, and the imprint that is then established, whether through infection or vaccination, will govern the response to variants for years afterward. This is not a hypothetical consideration. The choice of ancestral spike as the imprinting antigen in the 2020 to 2021 vaccination rollout committed the global population to an imprint that subsequent Omicron variants had to overcome, and updated boosters had to be designed against this consolidated imprint rather than against a naive substrate [171]. Preparedness planning should therefore treat the first vaccine product deployed against a novel pathogen not only as the tool for the first pandemic wave but also as the imprinting event for the subsequent decade, and the choice of that product should be evaluated with the durability of the imprint explicitly in mind.

**Table 3:** Imprint-aware vaccine design strategies and current development status**.** Strategies are grouped by the design lever they act on (immunogen engineering, schedule engineering, adjuvant selection, cohort-tailored deployment) as introduced in Section 7.1, Section 7.2, Section 7.3 and Section 7.4. Evidence tier reflects the highest-quality demonstration currently available for each strategy. Preclinical, animal or in-vitro mechanistic data. Phase 1 or Phase 2, human immunogenicity trials without efficacy endpoint. Licensed or Phase 3, human trials with clinical efficacy or licensure. AS01, adjuvant system 01; AS03, adjuvant system 03; cHA, chimeric hemagglutinin; HA, hemagglutinin; mRNA, messenger RNA; NHP, nonhuman primates; RBD, receptor-binding domain; RSV, respiratory syncytial virus.

Strategy	Mechanism of Imprint Modulation	Best Example	Evidence Tier	Key References
**IMMUNOGEN ENGINEERING**				
Chimeric hemagglutinin (cHA)	Rotates the immunodominant HA head between successive doses while preserving the conserved stalk. Repeated exposure to divergent heads dilutes head-focused recall and refocuses B-cell selection toward the shared subdominant stalk.	cHA prime–boost regimens (live-attenuated cH8/1N1 prime, AS03-adjuvanted cH5/1N1 boost). Phase 1 in adults 18 to 39 years, Phase 2 with adjuvanted split cHA.	Phase 1 and Phase 2 (human immunogenicity)	Krammer and Palese 2013 [172]; Nachbagauer et al. 2021 [43]; Folschweiller et al. 2022 [137]; Styles et al. 2025 [64]
Mosaic RBD nanoparticles	Co-displays heterologous RBDs on a single particle. Recruits cross-reactive naive B cells that recognize conserved motifs shared across displayed variants, bypassing the memory-dominant recall to any single strain.	Mosaic-8 and mosaic-8b displaying SARS-CoV-2 plus seven animal sarbecovirus RBDs. Broad protection in NHPs and mice against matched and mismatched sarbecoviruses.	Preclinical (NHP challenge)	Cohen et al. 2021 [135]; Cohen et al. 2022 (mosaic-8b) [140]
Hyperglycosylation and epitope resurfacing	Masks immunodominant variable epitopes with engineered N-linked glycans, redirecting the response toward conserved subdominant sites that were previously outcompeted.	Hyperglycosylated HA head constructs (seven N-glycans on the head); glycan-shielded SARS-CoV-2 RBD constructs.	Preclinical (mouse, in-vitro)	Bajic et al. 2019 [173]; Shinnakasu et al. 2021 [141]
**SCHEDULE ENGINEERING**				
Extended prime–boost interval	Lengthens the interval between doses to allow contraction of the memory compartment, reducing epitope masking by circulating antibody and giving naive B cells a competitive window to enter secondary germinal centers.	12 to 16 week interval between BNT162b2 doses in naive adults, versus the licensed 3 to 4 week schedule.	Observational cohort (human)	Tauzin et al. 2022 [146]; Parry et al. 2022 [147]; Payne et al. 2021 [142]
Heterologous prime–boost	Switches vaccine platforms between doses. Different innate signals and antigen-presentation kinetics recruit a broader B-cell repertoire and partially uncouple the recall from the initial imprint.	ChAdOx1 nCoV-19 prime followed by BNT162b2 boost (Com-COV). Higher IgG geometric mean and improved real-world protection versus homologous ChAdOx1/ChAdOx1.	Phase 2 and real-world cohort (human)	Liu et al. 2021 [143]; Stuart et al. 2022 [144]; Pozzetto et al. 2021 [145]
Variant-only monovalent boosting	Removes ancestral antigen from the formulation but does not prevent recall of ancestral-cross-reactive memory; variant-specific recruitment may increase after repeated variant exposure.	Monovalent XBB.1.5 and monovalent JN.1 boosters. Substantial GC-mediated broadening against BA.2.86 and JN.1 lineage.	Licensed (FDA and WHO recommendation)	Yisimayi et al. 2024 [19]; Tortorici et al. 2024 [92]; Johnston et al. 2024 [93]; Wang et al. 2024 [162]
**ADJUVANT SELECTION**				
AS03 (squalene emulsion + alpha-tocopherol)	Prolongs germinal-center activity, increases somatic hypermutation, and drives progressive accumulation of broadly neutralizing clones. Recruits naive B cells against novel epitopes while re-engaging memory for further affinity maturation.	AS03-adjuvanted CoVLP SARS-CoV-2 vaccine (progressive breadth over 6 months); AS03-adjuvanted cHA regimens (durable stalk-directed response).	Phase 2 and Phase 3 (human)	Grigoryan et al. 2024 [154]; Goll et al. 2022 [153]; Nachbagauer et al. 2021 [43]; O’Hagan et al. 2021 [152]
AS01 (liposomal MPL + QS-21)	Induces early IFN-gamma burst in the draining lymph node driving Tfh differentiation and sustained germinal centers. Compensates for immunosenescence-limited germinal-center capacity in older adults with deep imprints.	ZOE-50 enrolled adults aged ≥50 years; AReSVi-006 enrolled adults aged ≥60 years.	Licensed (Phase 3 efficacy)	Lal et al. 2015 [157]; Papi et al. 2023 [120]; Coccia et al. 2017 [156]; Roman et al. 2024 [155]
**COHORT-TAILORED DEPLOYMENT**				
Passive immunization in infants (nirsevimab)	Bridges the vulnerability window with long-acting monoclonal antibody without inducing B-cell memory. May defer natural infection without itself inducing B-cell memory; whether this changes the timing or quality of the later active imprint remains unknown.	Nirsevimab targeting the prefusion F conformation of RSV. HARMONIE Phase 3b: 83.2% efficacy against RSV hospitalization in infants.	Licensed (Phase 3 efficacy)	Drysdale et al. 2023 [107]
Maternal vaccination for infant protection	Delivers preformed prefusion-specific IgG across the placenta. Creates a prefusion-F-specific passive-antibody environment before first natural exposure; any effect on the infant’s later active imprint remains to be determined.	RSVpreF (Abrysvo) at 24 to 36 weeks gestation. MATISSE trial: 81.8% efficacy against severe RSV LRTI within 90 days postnatal.	Licensed (Phase 3 efficacy)	Kampmann et al. 2023 [4]; Simoes et al. 2022 (RSVpreF safety) [174]
High-dose or MF59-adjuvanted vaccines for older adults	Compensates for immunosenescence-limited germinal-center reactivity in adults with decades-old imprints. Preferentially recruits memory recall and increases magnitude when broadening is no longer achievable.	High-dose trivalent inactivated influenza vaccine (Fluzone HD); MF59-adjuvanted influenza vaccine (Fluad). ACIP preferential recommendation for adults 65+.	Licensed (Phase 3 efficacy)	Chit et al. 2015 [169]; Grohskopf et al. 2024 [159]
Cohort-specific product allocation	Aligns antigen design, platform and delivery route with the target birth-cohort’s imprint status. Requires functional immunization information systems and stratified cost-effectiveness analysis.	UK JCVI RSV program: maternal RSVpreF (Abrysvo) for pregnant persons plus the same RSVpreF product (Abrysvo) for adults aged 75–79 years. ACIP recommendation for a second dose of the 2024–2025 COVID-19 vaccine for adults aged ≥65 years.	Policy-implemented (national programs)	Groom et al. 2015 [167]; JCVI RSV recommendation 2023 [168]; MMWR 2024 [166]

## 8. Conclusions and Research Priorities

This review distinguishes the utility of cross-reactive memory from preferential first-antigen bias. Antigenic distance, conserved-epitope accessibility, and exposure interval may influence both, but available studies rarely isolate a protective effect attributable to the bias itself. The three-dimensional scheme used here is conceptual and hypothesis-generating; it has not been jointly fitted or independently validated, and it is not intended to predict imprinting outcomes ex ante. Recent mathematical models, particularly DynaVac, quantitatively formalize related variables within calibrated systems and therefore complement, rather than validate, this cross-pathogen organizational scheme [128,129,130,131]. Influenza data show that a history of childhood HA exposure is associated with later group-matched protection against H5N1 and H7N9 [15]. They do not establish that first-antigen preference is superior to an equivalent unbiased memory response. SARS-CoV-2 studies show that repeated variant exposure can broaden cross-reactive memory and expand de novo responses, while ancestral bias persists or diminishes. Imprinting should therefore be regarded as a potentially restrictive feature of immune history that vaccine design may mitigate, whereas protection after repeated exposure should be attributed to broadened memory unless an independent benefit of the bias is demonstrated.

Six research priorities are motivated by this hypothesis-generating conceptual scheme. First, longitudinal fine-needle aspiration studies of vaccinated humans, extending the approaches used by Turner and colleagues and Kim and colleagues, should be applied systematically across imprint states, age cohorts, and vaccine platforms to directly observe germinal-center reengagement and imprint modification in tissue rather than only in serum [160,161]. Second, preclinical animal models of long-interval heterologous prime–boost that recapitulate human memory dynamics, including realistic contraction, memory consolidation, and re-exposure intervals, are needed to move beyond the short prime–boost regimens that currently dominate the preclinical literature. Third, real-world epidemiological evidence on RSV imprint effects should be actively collected in the first birth cohorts covered by maternal RSVpreF and nirsevimab, from the 2023 to 2026 seasons through school entry, to determine whether the prefusion-F-directed passive-antibody environments created by maternal RSVpreF vaccination or infant nirsevimab influence subsequent active imprinting and later childhood RSV responses, as hypothesized above for RSV. Fourth, systematic head-to-head comparisons of adjuvant classes, including AS01, AS03, and MF59, should be undertaken in aged and previously imprinted populations to identify which platforms most efficiently broaden the response against a consolidated imprint. Fifth, prioritize deep mutational scanning of subdominant conserved epitopes on the HA stalk, the SARS-CoV-2 S2 subunit, and the RSV F site Ø under memory-antibody pressure to define which epitopes remain accessible when pre-existing antibodies mask the dominant sites and to inform the next generation of immunogen engineering. Sixth, prospectively specified datasets should jointly vary antigenic distance, dose, exposure interval, prior exposure sequence, and vaccine valency and should include separate training and external validation cohorts. Such datasets would enable direct comparison of calibrated models, such as DynaVac, with simpler qualitative hypotheses and would test whether zone boundaries transfer across platforms, populations, and pathogens without assuming they do [128].

The practical goal is not to erase immune memory but to preserve useful cross-reactive specificities, recruit responses to new epitopes, and minimize excessive first-antigen bias. Vaccines against novel pathogens should be evaluated for both the breadth of memory they establish and the extent to which that memory preferentially constrains subsequent responses. This distinction offers a conservative basis for designing durable, cross-protective vaccination strategies without assuming that imprinting bias is beneficial.

## Figures and Tables

**Table 1:** Key mechanistic and epidemiological studies of influenza immune imprinting. Selected studies that define the empirical foundation of influenza immune imprinting, from the original Francis observation to recent adjuvant-augmented chimeric hemagglutinin work. Rows are ordered chronologically. The Section column indicates where each study is discussed in the main text.

Study	Design	Cohort/Model (N)	Key Finding	Section	Ref.
Francis 1960	Historical seroepidemiology	US adults, multi-cohort HAI	First formalization of Original Antigenic Sin; response to influenza reinfection dominated by antibody to strain of first childhood exposure.	Cohort effects	[9]
Davenport, Hennessy, Francis 1953	Serological cross-sectional	US birth cohorts 1889–1952	Antigenic seniority: HAI titer hierarchy reflects order of encounter, not most recent exposure.	Cohort effects	[40]
Arevalo, McLean, Belongia, Cobey 2020	Modeling + surveillance serology	Wisconsin test-negative cohort 2007–2015	Earliest childhood infections predict age distribution of seasonal H1N1 vs. H3N2 cases decades later.	Cohort effects; pandemic risk	[8]
Gostic, Ambrose, Worobey, Lloyd-Smith 2016	Case–control epidemiology	H5N1 and H7N9 human cases, WHO-linked cohorts	Childhood HA-group exposure is associated with an approximately 75% lower risk of severe disease from same-group avian strains; the data support cross-reactive memory and do not distinguish it from demonstrated imprinting bias.	Pandemic risk	[15]
Gostic, Bridge, Brady, Viboud, Worobey, Lloyd-Smith 2019	Birth-year risk analysis	US seasonal influenza surveillance 1997–2018	Childhood imprint status is associated with birth-year-specific risk for both H1N1 and H3N2 across multiple seasons.	Pandemic risk	[17]
Worobey, Han, Rambaut 2014	Molecular phylogenetics	Historical 1918 pandemic H1N1 sequences	Establishes the group-1 versus group-2 HA phylogeny that underlies the birth-cohort imprint map.	Pandemic risk	[16]
Krammer, Pica, Hai, Margine, Palese 2013	Preclinical, mouse	Chimeric HA constructs (cH5/1, cH9/1)	Proof of concept that head-swap chimeric HA immunization redirects the response to the conserved stalk domain.	Repeat vaccination; immunogen engineering	[41]
Bernstein, Guptill, Naficy, Nachbagauer et al. 2021	Phase 1 randomized trial	Healthy adults 18–39 years, N = 65	Sequential chimeric HA vaccination is safe and induces broadly cross-reactive stalk antibodies in adults with prior H1 imprint.	Repeat vaccination; immunogen engineering	[42]
Nachbagauer, Feser, Naficy et al. 2021	Phase 1 randomized trial	Healthy adults 18–39 years, N = 65	AS03-adjuvanted inactivated chimeric HA boost after live-attenuated prime induces broad and long-lasting cross-reactive stalk antibodies.	Repeat vaccination; immunogen engineering	[43]
Skowronski, Chambers, De Serres et al. 2017	Test-negative case–control, multi-season	Canadian Sentinel Practitioner Network, 2010–2015 seasons	Repeated annual vaccination attenuates vaccine effectiveness against A(H3N2), consistent with negative interference from prior-season vaccination.	Repeat vaccination	[44]
Akhtar, Styles, Gu et al. 2025	Preclinical, nonhuman primates	Rhesus macaques, AS03-adjuvanted chimeric HA vaccine	AS03 amplifies imprint-broadening: adjuvanted chimeric HA induces long-lived stalk-specific plasma cells in bone marrow and lymph nodes for more than one year (through week 94, i.e., 15 months after cH5/1N1 immunization, sustaining cross-reactive protection.	Repeat vaccination; adjuvant selection	[45]

**Table 2:** Key mechanistic studies of SARS-CoV-2 immune imprinting. Studies are grouped by imprinting scenario (restrictive first-antigen bias/mitigation of bias/protective cross-reactive memory despite persistent bias). Columns summarize cohort or model, exposure history, key mechanistic finding, and interpretation within the proposed imprinting framework. DMS, deep mutational scanning; GC, germinal center; mAb, monoclonal antibody; NAb, neutralizing antibody; bnAb, broadly neutralizing antibody; RBD, receptor-binding domain; NT50, 50% neutralization titer.

Study (ref)	Cohort/Model	Exposure History	Key Mechanistic Finding	Interpretation for Imprinting
**IMPRINTING HARMS**				
Aydillo et al. 2021 [18]	Adults with prior seasonal HCoV exposure, hospitalized with COVID-19	Primary SARS-CoV-2 infection in seropositive adults	Antibody response dominated by cross-reactive HCoV memory. De novo SARS-CoV-2-specific antibodies were reduced compared to naive individuals.	Earliest direct human evidence that imprinting can operate across coronavirus species. Pre-existing HCoV memory constrains the response to a new betacoronavirus.
Park et al. 2022 [38]	BNT162b2-vaccinated adults with breakthrough Omicron BA.1 infection	Two or three ancestral mRNA doses plus BA.1 breakthrough	Post-breakthrough plasmablasts predominantly recalled ancestral memory. BA.1-specific de novo antibodies were rare. Serum neutralization of BA.4 and BA.5 remained lower than of ancestral.	Ancestral imprinting persists after heterologous exposure. Memory recall from vaccination outcompetes naive engagement even during active variant infection.
Reynolds et al. 2022 [76]	UK healthcare workers, longitudinal cohort	Two or three ancestral vaccine doses plus Omicron BA.1 breakthrough	BA.1 breakthrough in triple-vaccinated individuals boosted ancestral-reactive antibodies but not Omicron-specific ones. Repeated ancestral priming reduced the breadth of the recalled response.	Ancestral-spike imprinting is evident in SARS-CoV-2 boosting: repeated ancestral priming before variant exposure is associated with a narrower recalled response.
Wang, Q. et al. 2023 [80]	Adults who received bivalent WT/BA.5 booster	Three ancestral mRNA doses plus bivalent WT/BA.5 booster	Bivalent booster elicited nearly identical NT50 titers as monovalent ancestral booster. BA.5-specific B-cell response was minimal.	Deep imprinting (as termed by the authors). Adding a variant antigen to an ancestral platform does not overcome memory dominance when B cells have been repeatedly primed with ancestral spike.
**IMPRINTING PARTIALLY MITIGATED (“OVERRIDE”)**				
Yisimayi et al. 2024 [19]	Adults with multiple Omicron exposures (infection or vaccination), n = 250	Repeated Omicron exposures (BA.1 or BA.5 vaccination plus BA.5/BF.7 breakthrough)	After two Omicron exposures, the ancestral-cross-reactive fraction of the response dropped from >50% to ~20%. Omicron-specific naive B cells were recruited into germinal centers and produced BA.2.86- and JN.1-neutralizing antibodies.	Ancestral imprinting can be partially overridden, operationally meaning substantially mitigated rather than completely eliminated. The mechanism requires two or more Omicron-only exposures separated in time, allowing new GCs to seed a naive-derived response.
Alsoussi et al. 2023 [12]	Adults vaccinated with mRNA-1273.222 (bivalent WT/BA.5) booster, lymph node biopsies	Three ancestral mRNA doses plus bivalent booster	GC B cells six weeks after boost showed clonal turnover. Naive-derived Omicron-reactive clones entered GCs, though ancestral-derived clones still dominated numerically.	GC dynamics permit gradual mitigation even with ancestral-containing boosters. Greater mitigation is favored by sustained variant exposure without ancestral-antigen competition.
Tamura et al. 2024 [94] Dadonaite et al. 2024 [82]	Hamster infection model plus full-spike deep mutational scanning libraries (XBB.1.5 and BA.2 backgrounds) profiled against polyclonal human sera	Syrian hamsters infected with BA.2.86 versus BA.2; human sera from XBB*-infected vaccinees	BA.2.86 showed spike fusogenicity comparable to BA.2 but significantly lower intrinsic pathogenicity and lower growth kinetics in vitro and in vivo; full-spike deep mutational scanning of XBB.1.5 and BA.2 libraries covered nearly all spike mutations present in BA.2.86-descended clades and mapped serum escape predominantly to RBD sites, providing the mutation-level basis for the antigenic distance of BA.2.86 from earlier Omicron lineages	Antigenic novelty of BA.2.86 is driven by accumulated spike substitutions rather than by attenuated recall of ancestral cross-reactive memory; the animal data do not address imprinting and should not be read as evidence of override.
**CROSS-REACTIVE MEMORY HELPS DESPITE PERSISTENT IMPRINTING**				
Pinto et al. 2021 [84]	Human monoclonal antibody S2P6 isolated from a SARS-CoV-2 convalescent donor	Prior ancestral SARS-CoV-2 infection	S2P6 binds the S2 stem helix (residues 1146–1159), a highly conserved epitope. It neutralizes SARS-CoV-2, SARS-CoV, MERS-CoV, and multiple bat sarbecoviruses.	Ancestral priming can elicit clones targeting conserved S2 epitopes. These clones are recalled and reinforced by variant boosters; without an unbiased-memory comparator this does not demonstrate that preferential ancestral.
Tortorici et al. 2021 [86]	Human monoclonal antibody S2X259 from SARS-CoV-2 convalescent donor	Prior ancestral SARS-CoV-2 infection	S2X259 binds the class 4 RBD epitope (T376, R408, S503). It neutralizes every tested sarbecovirus clade and shows high genetic barrier to escape.	Cross-sarbecovirus protection is achievable from ancestral priming when memory targets conserved class 4 RBD sites. Ancestral priming can establish cross-reactive memory relevant to pan-sarbecovirus vaccine design, although the preferential ancestral bias itself has not been shown to be beneficial.
Schaefer-Babajew et al. 2023 [78]	Adults receiving mRNA vaccination with or without prior monoclonal antibody infusion	Prior anti-spike mAb plus mRNA vaccination	Passively administered anti-spike antibody masked immunodominant epitopes and redirected the memory response toward conserved subdominant sites. Back-boosting to conserved epitopes was enhanced.	Antibody feedback (epitope masking) can be exploited therapeutically. Masking of dominant sites can redirect pre-existing memory toward cross-reactive breadth; this does not demonstrate a benefit of preferential ancestral bias.
Inoue et al. 2023 [79]	BNT162b2-vaccinated adults, lymph node biopsy, longitudinal follow-up	Two ancestral mRNA doses plus Omicron BA.1 breakthrough infection	Antibody feedback from vaccination-induced memory facilitated the development of Omicron-reactive memory B cells in GCs. Cross-reactive clones expanded through iterative GC cycles.	Ancestral-primed cross-reactive memory can act as a scaffold even while preferential ancestral bias persists. Memory recall combined with GC persistence enables gradual broadening rather than pure epitope lock-in.
Malladi et al. 2025 [13]	Adults receiving a bivalent ancestral/BA.4-5 mRNA booster, with longitudinal lymph-node sampling and B-cell repertoire analysis	Prior ancestral immunity plus a bivalent ancestral/BA.4-5 mRNA booster	GC-mediated broadening was observed after a bivalent ancestral/BA.4-5 booster: recalled memory B cells re-entered germinal centers and acquired extensive somatic hypermutation, resulting in increased cross-variant neutralization breadth.	Variant boosting can broaden an imprinted response through continued germinal-center maturation of recalled memory B cells, even when the ancestral antigen remains in the vaccine formulation.

## Data Availability

No new data were created or analyzed in this study. Data sharing is not applicable to this article.

## Abbreviations

The following abbreviations are used in this manuscript:

Ab	antibody
ACIP	Advisory Committee on Immunization Practices
AReSVi-006	phase 3 trial of RSVPreF3 in older adults
AS01	adjuvant system 01 (GlaxoSmithKline)
AS03	adjuvant system 03 (GlaxoSmithKline)
BA.1	SARS-CoV-2 Omicron sublineage BA.1
BA.2	SARS-CoV-2 Omicron sublineage BA.2
BA.2.86	SARS-CoV-2 Omicron sublineage BA.2.86
BA.4	SARS-CoV-2 Omicron sublineage BA.4
BA.5	SARS-CoV-2 Omicron sublineage BA.5
BCR	B-cell receptor
bnAb(s)	broadly neutralizing antibody(ies)
BNT162b2	Pfizer-BioNTech COVID-19 mRNA vaccine
CDC	US Centers for Disease Control and Prevention
ChAdOx1	chimpanzee adenovirus Oxford 1 (viral vector)
CIDRAP	Center for Infectious Disease Research and Policy
COBRA	computationally optimized broadly reactive antigen
Com-COV	Comparing COVID-19 Vaccine Schedule Combinations trial
Com-COV2	Comparing COVID-19 Vaccine Schedule Combinations trial 2
COVID-19	coronavirus disease 2019
cryo-EM	cryogenic electron microscopy
F	fusion protein (RSV surface glycoprotein)
FDA	US Food and Drug Administration
FDC	follicular dendritic cell
FI-RSV	formalin-inactivated respiratory syncytial virus vaccine
G	attachment glycoprotein (RSV surface protein)
GC(s)	germinal center(s)
H1	hemagglutinin subtype 1
H1N1	influenza A hemagglutinin 1, neuraminidase 1
H2	hemagglutinin subtype 2
H2N2	influenza A hemagglutinin 2, neuraminidase 2
H3	hemagglutinin subtype 3
H3N2	influenza A hemagglutinin 3, neuraminidase 2
H5	hemagglutinin subtype 5
H5N1	influenza A hemagglutinin 5, neuraminidase 1
H7	hemagglutinin subtype 7
H7N9	influenza A hemagglutinin 7, neuraminidase 9
HA	hemagglutinin
HAI	hemagglutination inhibition
HARMONIE	nirsevimab post-authorization real-world effectiveness study
IBV	influenza B virus
IgG	immunoglobulin G
IgM	immunoglobulin M
JN.1	SARS-CoV-2 Omicron sublineage JN.1
KP.2	SARS-CoV-2 Omicron sublineage KP.2
KP.3	SARS-CoV-2 Omicron sublineage KP.3
LAIV	live attenuated influenza vaccine
mAb(s)	monoclonal antibody(ies)
MATISSE	phase 3 trial of RSVpreF maternal vaccine
MELODY	phase 3 trial of nirsevimab in healthy term and late-preterm infants
MERS-CoV	Middle East respiratory syndrome coronavirus
MF59	squalene-based oil-in-water adjuvant
MMWR	Morbidity and Mortality Weekly Report (US CDC)
mRNA	messenger RNA
NA	neuraminidase
NK	natural killer (cell)
OAS	original antigenic sin
post-F	postfusion conformation of RSV F protein
pre-F	prefusion conformation of RSV F protein
RBD	receptor-binding domain (of SARS-CoV-2 spike)
RSV	respiratory syncytial virus
RSV-A	respiratory syncytial virus subgroup A
RSV-B	respiratory syncytial virus subgroup B
RSVpreF	bivalent RSV prefusion F vaccine (Pfizer, Abrysvo)
RSVPreF3	RSV prefusion F vaccine (GlaxoSmithKline, Arexvy)
S	spike protein (of SARS-CoV-2)
S1	spike subunit 1
S2	spike subunit 2
S2P6	human monoclonal antibody targeting the conserved S2 stem
SARS	severe acute respiratory syndrome
SARS-CoV	severe acute respiratory syndrome coronavirus
SARS-CoV-2	severe acute respiratory syndrome coronavirus 2
SHM	somatic hypermutation
Tfh	T follicular helper (cell)
Th1	T helper type 1
Th2	T helper type 2
UK	United Kingdom
US	United States
VE	vaccine effectiveness
VLP	virus-like particle
WHO	World Health Organization
WT	wild type
Wuhan-Hu-1	ancestral SARS-CoV-2 reference sequence
XBB	SARS-CoV-2 recombinant Omicron lineage XBB
XBB.1.5	SARS-CoV-2 Omicron sublineage XBB.1.5
XEC	SARS-CoV-2 Omicron recombinant sublineage XEC
ZOE-50	phase 3 trial of the adjuvanted zoster subunit vaccine
